# Traditional Chinese medicine Euodiae Fructus: botany, traditional use, phytochemistry, pharmacology, toxicity and quality control

**DOI:** 10.1007/s13659-023-00369-0

**Published:** 2023-02-15

**Authors:** Si-Jia Xiao, Xi-Ke Xu, Wei Chen, Jia-Yun Xin, Wen-Lin Yuan, Xian-Peng Zu, Yun-Heng Shen

**Affiliations:** 1 Department of Natural Medicinal Chemistry, School of Pharmacy, Naval Medical University, No. 325 Guohe Road, Yangpu District, Shanghai, 200433 China; 2 School of Pharmacy, Shandong University of Traditional Chinese Medicine, Jinan, 250355 China

**Keywords:** Euodiae Fructus, Traditional uses, Phytochemistry, Pharmacology, Toxicology

## Abstract

Euodiae Fructus, referred to as “Wuzhuyu” in Chinese, has been used as local and traditional herbal medicines in many regions, especially in China, Japan and Korea, for the treatment of gastrointestinal disorders, headache, emesis, aphtha, dermatophytosis, dysentery, etc. Substantial investigations into their chemical and pharmacological properties have been performed. Recently, interest in this plant has been focused on the different structural types of alkaloids like evodiamine, rutaecarpine, dehydroevodiamine and 1-methyl-2-undecyl-4(1H)-quinolone, which exhibit a wide range of pharmacological activities in preclinical models, such as anticancer, antibacterial, anti-inflammatory, anti-cardiovascular disease, etc. This review summarizes the up-to-date and comprehensive information concerning the botany, traditional uses, phytochemistry, pharmacology of Euodiae Fructus together with the toxicology and quality control, and discusses the possible direction and scope for future research on this plant.

## Introduction

Euodiae Fructus (EF), known as “Wuzhuyu” in China, “Goshuyu” in Japan and “Osuyu” in Korea, are the dried and nearly ripe fruits of *Euodia rutaecarpa* (Juss.) Benth. (ER), *E. rutaecarpa* (Juss.) Benth var. *officinalis* (Dode) Huang (ERO), and *E. rutaecarpa* (Juss.) Benth. var. *bodinieri* (Dode) Huang (ERB). It has been used as traditional Chinese medicine (TCM) for more than 2000 years and is officially listed in multiple versions of Chinese Pharmacopoeia. At the same time, it is also traditionally and ethnically used in Japan and Korea. According to the records of TCM, Euodiae Fructus could be widely used either alone or in combination with other herbal medicines as remedies for gastrointestinal disorders (abdominal pain, dysentery), headache, emesis, aphtha, dermatophytosis, dysentery, amenorrhoea, menorrhalgia and postpartum haemorrhage. However, it is worth noting that irrational use of this herb could cause toxic symptoms such as stomach ache, vomiting, blurred vision, etc.

**Fig. 1:**
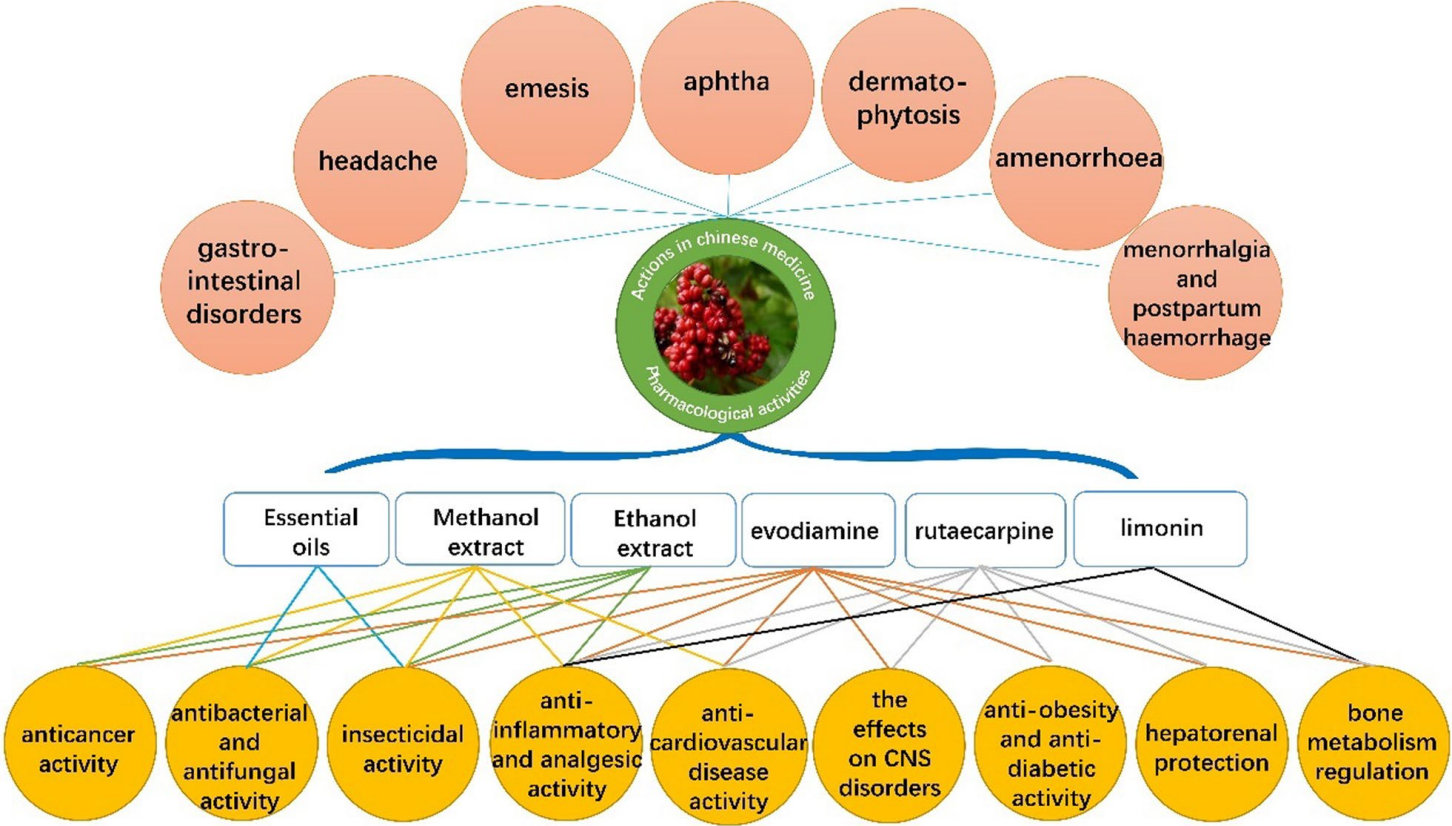
Multiple pharmacological effects of Euodiae Fructus

With the increasing interest paid to the pharmacologically phytochemicals from the Euodiae Fructus, a lot of investigations related to the phytochemical and pharmacological aspects of this plant have been carried out. To date, a variety of chemical constituents, including alkaloids, terpenoids and steroids, as well as phenols and volatile oils, have been isolated and identified from Euodiae Fructus. Pharmacological studies revealed that the crude extracts and purified compound possess a wide spectrum of biological activities, involving in anticancer, antibacterial, anti-inflammatory, insecticide, anti-cardiovascular, neuroprotective, anti-obesity and anti-diabetic activities, confirmed by various in vivo and in vitro experiments, as shown in Fig. 1. In recent years, several reviews have been published on the chemical and biological activities of ivodimine [1, 2], erythrartine [3, 4] and citrinin [5]. A review of Euodiae Fructus is essential for present and future study toward improving phytochemical and pharmacological investigation. Herein, we systematically described and summarized the study advances of Euodiae Fructus in recent decades, including phytochemical, pharmacological effects, toxicity, and quality control. We reviewed the literature up to February 2021.

## Botanical descriptions

In Chinese Pharmacopoeia, the dried and nearly ripe fruits of three plants of the genus *Euodia rutaecarpa* (Juss.) Benth. (ER), *E. rutaecarpa* (Juss.) Benth var. *officinalis* (Dode) Huang (ERO), and *E. rutaecarpa* (Juss.) Benth. var. *bodinieri* (Dode) Huang (ERB) are commonly known as Euodiae Fructus.

The common botanical characteristics of the above three plants are small trees or shrubs, 3–5 m high. They often have opposite odd-pinnate leaves. Their inflorescences are terminal; the flowers of the male inflorescence are separated from each other, and the flowers of the female inflorescence are dense or separated. The dioecious flowers have 4 or 5 sepals, petals, stamens, and carpels. The fruits are oblate and split into 5 petals when mature, and they are follicle-like, purplish red, with large oil glands on the surface, and each fruit has one seed, black and shiny. The flowering period ranges from June to August, and the fruit period is typically from August to November. However, there are also some differences of them in botanical descriptions and distribution area, as shown in Table 1.

**Table 1:** The differences in botanical descriptions between three plants

Plant	Botanical description differences	Distribution area
*Euodia rutaecarpa* (Juss.) Benth	Its leaflets are larger, up to 7 cm wide, and slightly thicker than paper, and both sides are densely hairy; its rachis of the inflorescence are reddish-brown hairy, and the female inflorescence has denser flowers, with petals up to 5 mm long and densely hairy on the inner surface.; its fruit stalk is shorter and stout	It is mainly distributed in Guangdong, Guangxi and southern Yunnan
*Euodia rutaecarpa* (Juss.) Benth var. *officinalis* (Dode) Huang	Its leaflets are like paper, more than 5 cm wide. The back of the leaf is densely covered with long hairs with large oil spots. There are fewer fruits on the infructescence, dense or loose with each other	It is mainly distributed in Zhejiang, Jiangsu and Jiangxi
*Euodia rutaecarpa* (Juss.) Benth. var. *bodinieri* (Dode) Huang	Its leaflets are slightly thinner than paper, and only the veins on the back of the leaf are sparsely pilose. The flowers on the female inflorescence are separated from each other, the petals are about 4 mm long, and the inner surface is sparsely or almost glabrous; its fruit stalks are slender and elongated	It is mainly distributed in northern Guangdong, northeastern Guangxi, southwestern Hunan, and southeastern Guizhou

These three plants usually grow in mountains, roadsides, or sparse forests. It is mainly produced in the southern regions of China (such as Hunan, Guizhou, Sichuan, Yunnan), as well as in Japan, Korea, Bhutan, northeast India, Myanmar, and Nepal.

## Traditional uses

Euodiae Fructus has a long history as a traditional remedy and has been widely used Chinese medicine as recorded in the ancient herbal books and Pharmacopoeia of the People’s Republic of China (Editorial Committee of Chinese Pharmacopoeia, 2020). According to the history of TCM, Euodiae Fructus, initially recorded in "Shen Nong’s Herbal Classic", is listed as a middle-grade herbal item and also described as being pungent and bitter in taste and can return to the liver, spleen, stomach and kidney meridians. According to the records of “Ri Hua Zi Ben Cao”, it could strengthen the spleen, treat abdominal pain, beriberi, edema, and postpartum haemorrhage. Furthermore, it was found to kill harmful insects and prevent tooth decay in “Ben Cao Shi Yi”. As it was recorded in “Compendium of Materia Medica”, the main function of Euodiae Fructus was to improve digestion, relieve headache, abdominal pain and treat hemorrhoids in throat, mouth and tongue [6]. According to the 2020 Edition of Chinese Pharmacopoeia, Euodiae Fructus is often used for external use and the recommended dosage is 2–5 g, the “standard” processing method of Euodiae Fructus is stir-frying with licorice water extract, other usual processing methods include washing with hot or cold water [7].

Since the compatibility of medicines is considered to improve effects, reduce toxicity, or achieve synergistic or balanced effects [7]. Euodiae Fructus was often combined with Jujubae Fructus, which has the effect of treating stomachache and pregnancy headache. If combined with Angelicae Sinensis Radix, it could promote blood circulation and relieve menstrual pain. When combined with Zingiberis Rhizoma Recens, it could promote yang and dispel cold. Moreover, Euodiae Fructus could be used in a combination with Codonopsis Radix or Foeniculi Fructus, thereby playing a significant role in tonifying and warming stomach, etc. Based on the above compatibility, Euodiae Fructus was typically used in polyherbal formulations in TCM (http://www.zysj.com.cn/zhongyaofang/index.html), and the composition and therapeutic effects of typical polyherbal formulations are summarized in Table 2. In recent years, numerous studies in vitro and vivo have indicated that “Zuo Jin Wan” (ZJW) possess good pharmacological effects, such as anti-inflammation, anti-ulcer [8], anti-acid [9], antidepressant-like [10], and anti-cancer properties [11]. Noteworthy, Li et al. conducted a systematic review and meta-analysis according to a total of 1736 patients in 18 studies, indicating “Wenjing Tang” was shown to be significantly superior to nonsteroidal anti-inflammatory drugs in improving primary dysmenorrhea in terms of clinical effective rate, the visual analogue scale, and the pain scale for dysmenorrhea [12].

**Table 2:** Typical formulas and prescriptions in traditional Chinese medicine including Euodiae Fructus

Formula/Preparation name	Composition	Traditional and clinical use	References
Wuzhuyu Tang	**Evodiae Fructus**, *Ginseng Radix et Rhizoma*, Zingiberis Rhizoma Recens, *Jujubae Fructus*	Treating for epigastrium distension, vomiting, habitual migraine, paroxysmal headache, hiccups, feeling of cold hands and feet	Treatise on Cold Pathogenic Diseases, 1066
Zuo Jin Wan	*Coptidis Rhizoma*, **Euodiae Fructus**	Treating for esophagitis, gastritis, duodenal ulcer	Chinese Pharmacopoeia, 2020, P. 802–803
Wenjing Tang	**Euodiae Fructus**, *Angelicae Sinensis Radix*, *Chuanxiong Rhizoma*, *Paeoniae Radix Alba*, *Ginseng Radix et Rhizoma*, etc	Treating for acitvating blood circulation and relieving blood stasis, amenorrhea and irregular menstration	Essential Prescriptions from the Golden Cabinet, 1066
Wu Ji Wan	*Coptidis Rhizoma*, **Euodiae Fructus**, *Paeoniae Radix Alba*	Treating for burning pain in epigastric, vomiting and swallowing acid, bitter taste in mouth, abdominal pain and diarrhea	Chinese Pharmacopoeia, 2020, P. 810
Sishen Wan	*Myristicae Semen*, *Psoraleae Fructus*, *Schisandrae Chinensis Fructus*, *Jujubae Fructus*, **Euodiae Fructus**	Treating for chronic diarrhea and intestinal tuberculosis	Chinese Pharmacopoeia, 2020, P. 829–830
Huatuo Zaizao Wan	Concentrated water-honeyed pill composed of *Chuangxiong Rhizoma*, **Euodiae Fructus**, *Borneolum Syntheticum*	Treating for activating blood circulation, resolving phlegm, and stroke rehabilitation	Chinese Pharmacopoeia, 2020, P. 894
Changkang Pill	Berberine hydrochloride, *Aucklandiae Radix*, **Euodiae Fructus**	Treating for diarrhea and dysentery	Chinese Pharmacopoeia, 2020, P. 1052–1053
Ai Fu Nuan Gong Wan	*Artemisiae Argyi Folium*, *Cyperi Rhizoma*, **Euodiae Fructus**, etc	Treating for irregular menstruation, dysmenorrhea, acyesis	Chinese Pharmacopoeia, 2020, P. 798

Besides, Euodiae Fructus is also popular in Japan and South Korea. According to Dongui Bogam, a representative Korean Medicine book, Euodiae Fructus has been frequently used as a prescription for treating headache, abdominal pain, vomiting, cold, reducing blood circulation and gynecological diseases (amenorrhea), with a dose of 2–8 g. It is also one of the main components of traditional herbal prescriptions for the treatment of sterility caused by irregular menstruation such as Chokyungjongok-Tang, Nangungjongsa-whan, and Onkyung-Tang [13]. In addition, Euodiae Fructus was introduced in Japan as early as Edo, mainly applied for the treatment of cold and pain. For example, Goshuyuto, a representative traditional Japanese medicine, also known as “Wuzhuyu Tang” in China and “Osuyu-tang” in Korea, is composed of four medicinal herbs, Euodiae Fructus, Ginseng Radix Et Rhizoma, Zingiberis Rhizoma Recens, Jujubae Fructus, and it could be used to treat migraine headache, nausea, beriberi, and heart failure [14].

## Phytochemistry

To date, more than 240 kinds of constituents have been isolated and identified from Euodiae Fructus, including 133 alkaloids, 36 terpenoids, 5 steroids, 51 phenols and 15 other compounds. Among them, alkaloids and terpenoids have been identified as the characteristic components. All compounds are summarized and compiled in Table 3.

**Table 3:** The compounds isolated from Euodiae Fructus and their activities

Compounds		Species	Biological activity	Pharmacological detail	References
Alkaloids					
1	Evodiamine	ER, ERB, ERO			[15]
2	Hydroxyevodiamine	ER			[16]
3	Carboxyevodiamine	ER			[16]
4	Acetonylevodiamine	ER			[17]
5	Dihydrorutaecarpine	ER			[18]
6	14-Formyl dihydrorutaecarpine	ER	Pro-inflammatory activities	Inhibited fMLP/CB-induced elastase release with IC_50_ of 48.8 μM	[19]
7	13*β*-Hydroxy Methylevodiamine	ER			[20]
8	Rutaecarpine	ER, ERB, ERO			[19]
9	1-Hydroxy-rutaecarpine	ER, ERO	Antitumor activities	Showed cytotoxic activities against HL60 and N-87 with IC_50_ values of 10.1 and 8.38 μM, respectively	[20, 21]
10	3-Hydoxyrutaecarpine	ER	Antitumor activities	Showed cytotoxic activities against HL60 with GI_50_ of 11.94 ± 2.00 μM	[20]
			*α*-glucosidase inhibitor	Showed moderate inhibitory effects against *α*-glucosidase, with IC_50_ values of 8.7 μM	[22]
11	7*β*-Hydroxy-rutaecarpine	ER, ERO	Antitumor activities	Showed cytotoxic activities against HL60 and N-87 with IC_50_ of 10.1 and 23.2 μM, respectively	[21]
			Antibacterial activity	Showed moderate inhibitory effects against *Bacillus cereus* with MIC value of 25 μM	[22]
12	10-Hydroxy-rutaecarpine	ER	P450 inhibitor	Decreased CYP1A1, CYP1A2, and CYP1B1 activities with respective IC_50_ values of 2.56 ± 0.04, 2.57 ± 0.11, and 0.09 ± 0.01 μM	[23]
13	(7*R*,8*S*)-7,8-Dihydroxy-rutaecarpine	ER	Antitumor activities	Showed cytotoxic activities against HL60 and N-87 with IC_50_ of 13.7 and 14.1 μM, respectively	[21]
14	(7*R*,8*S*)-7-Hydroxy-8-methoxy-rutaecarpine	ER	Antitumor activities	Showed cytotoxic activities against HL60 and N-87 with IC_50_ of 7.82 and 22.3 μM, respectively	[21]
15	(7*R*,8*S*)-7-Hydroxy-8-ethoxy-rutaecarpine	ER	Antitumor activities	Showed cytotoxic activities against HL60 and N-87 with IC_50_ of 8.31 and 27.9 μM, respectively	[21]
16	Hortiacine	ER			[20]
17	Rutaecarpine-10-*O*-*β*-D-Glucopyranoside	ER			[23]
18	Rutaecarpine-10-*O*-Rutinoside	ER			[23]
19	Dehydroevodiamine	ER, ERO			[24]
20	Evodiamide	ER			[25]
21	*N*-(2-methylarninobenzoyl) tryptarnine	ER	Antitumor activities	Showed cytotoxic activities against HL60 with GI_50_ of 57.43 ± 4.21 μM	[20]
22	Evodianinine	ER			[26]
23	Dievodiamine	ER			[27]
24	Rhetsinine	ER	Anti-diabetic activity	Inhibited aldose reductase with IC_50_ value of 24.1* μM* and inhibited sorbitol accumulation by 79.3% at 100 μM	[28]
			Insecticidal activity	Exhibited inhibition against *Xanthomonas oryzae* pv. *oryzicola*, and *Xanthomonas campestris* pv. *campestris*, with respective EC_50_ values of 3.13, 14.32, and 32.72 nmol	[29]
25	Goshuyuamide I	ER	Antitumor activities	Showed cytotoxic activities against HL60 with GI_50_ of 13.62 ± 1.10 μM	[20]
26	Goshuyuamide II	ER	Antitumor activities	Showed cytotoxic activities against HL60 with GI_50_ of 31.39 ± 3.21 μM	[20]
			*α*-Glucosidase inhibitor	Showed moderate inhibitory effects against *α*-glucosidase, with IC_50_ values of 22.1* μM*	[22]
27	Wuchuyuamide I	ER, ERO	Antitumor activities	Showed cytotoxic activities against HL60 and N-87 with IC_50_ of 15.1 and 20.1 μM, respectively	[21]
			Insecticidal activity	Possessed nematocidal activity against *Meloidogyne incognita* with LC_50_ values of 147.87 μg/mL; exhibited strong larvicidal activity against the early fourth instar larvae of *Aedes albopictus* with LC_50_ values of 26.16 μg/mL	[30, 31]
28	Wuchuyuamide II	ER			[32]
29	Wuchuyuamide III	ERO	Anticancer activity	Showed toxicity against HeLa and HT1080 cells with IC_50_ of 31.32 and 24.51 μM respectively	[33]
30	Wuchuyuamide IV	ERO	Anticancer activity	Showed toxicity against HeLa and HT1080 cells with IC_50_ of 31.91 and 24.52 μM respectively	[34]
31	Wuzhuyurutine A	ER			[35]
32	Wuzhuyurutine B	ER	Intestinal transport capacity	Demonstrated higher-level intestinal transcellular efflux at 5 μM	[36]
33	Wuzhuyurutine C	ER	Antitumor activities	Showed cytotoxic activities against HL60 with GI_50_ of 70.08 ± 1.56 μM	[20]
34	Wuzhuyurutine D	ER	Antitumor activities	Showed toxicity against HL60 and PC-3 with GI_50_ of 24.88 and 46.50 μM, respectively	[20]
35	Bouchardatine	ER	Antitumor activities	Showed cytotoxic activities against HL60 with GI_50_ of 71.88 ± 6.13 μM	[20]
36	Evollionine A	ER			[37]
37	Evollionine B	ER			[37]
38	Evollionine C	ER			[37]
39	*β*-Carboline	ER			[38]
40	1,2,3,4-Tetrahydro-1-oxo-carboline	ER			[20]
41	6-Methoxy-N-methyl-1,2,3,4-tetrahydro-*β*-carboline	ER			[39]
42	Evodiagenine	ER			[27]
43	(–)-Evodiakine	ER			[40]
44	( +)-Evodiakine	ER			[40]
45	3-Hydroxyacetylindole	ER			[41]
46	*N*-methyltryptamine	ER			[39]
47	*N*,*N*-Dimethyltryptamine	ER	Effect on 5-HT_1A_ receptor	Interacted with 5-HT_1A_ receptors with K_i_ values of 0.41 µM	[42]
48	5-Methoxy-*N*-methyltryptamine	ER			[39]
49	5-Methoxy-*N*,*N*-dimethyltryptamine	ER	Effect on 5-HT_1A_ receptors	Interacted with 5-HT_1A_ receptors with K_i_ values of 28 nM	[42]
50	10-Methoxygoshuyuamide II	ER	α-glucosidase inhibitor	Showed moderate inhibitory effects against *α*-glucosidase, with IC_50_ values of 23.9	[22]
			Antitumor activities	Displayed moderate inhibitory effect against four human cancer cell lines (MCF-7, Hepg-2, A549, and SHSY-5Y) with IC_50_ of 24.7 − 65.2 μM	[22]
			Antibacterial activity	Showed moderate inhibitory effects against *Bacillus cereus* with MIC values of 50 μM	[22]
51	(*S*)-7-Hydroxysecorutaecarpine	ER			[22]
52	Evodamide A	ER			[22]
53	13,14-Dihydrorutecarpine	ER			[22]
54	1-Methyl-2-ethyl-4(1*H*)-quinolone	ER			[43]
55	1-Methyl-2-(2-cyclopentylethyl)-4(1*H*)-quinolinone	ER			[44]
56	1-Methyl-2-pentyl-4-(1*H*)-quinolone	ER			[43]
57	1-Methyl-2-heptyl-4(1*H*)-quinolone	ER			[43]
58	1-Methyl-2-octyl-4(1*H*)- quinolone	ER	Antitumor activities	Showed cytotoxic activities against HL60 with GI_50_ of 21.04 ± 0.50 μM	[20]
59	1-Methyl-2-nonyl-4(1*H*)-quinolone	ER, ERO	NFAT and NF-кB inhibitor	Showed inhibitory effects against NFAT and NF-кB activity with IC_50_ value of 15.91 and 10.32 ± 0.69 μM, respectively	[45]
			Leukotriene biosynthesis inhibitors	Inhibited leukotriene biosynthesis in a bioassay using human polymorphonuclear granulocytes with IC_50_ of 12.1 μM	[46]
			Antitumor activity	Inhibited proliferation of human tumor lines HL-60, N-87, H-460, and Hep G2 cells with IC_50_ of 21.3, 23.3, 25.15 and 21.92 μM, respectively	[47]
			MAO-B inhibitor	Inhibited MAO activity dose-dependently with IC_50_ values of 2.3 μM	[48]
			Antibacterial activity	Against methicillin-resistant *Staphylococcus aureus* with MIC of 64 μg/mL; Against *Staphyloccocus epidermidis* ATCC12228, and *Bacillus subtilis* ATCC6633 with MIC of 8 and 16 μg/mL, respectively	[43, 49]
60	1-Methyl-2-[(*Z*)-4-nonenyl]-4(1*H*)-quinolone	ER	Antitumor activities	Inhibited proliferation of human tumor lines HL-60, N-87, H-460 and HepG2 cells with IC_50_ of 21.67, 17.25, 18.56 and 21.76 μM, respectively	[47]
61	1-Methyl-2-decyl-4(1*H*)-quinolone	ER	Antitumor activities	Inhibited proliferation of human tumor lines HL-60, N-87, H-460, and Hep G2 cells with IC_50_ values of 22.97, 21.69, 21.92 and 18.14 μM, respectively	[20, 47]
62	1-Methyl-2-undecyl-4(1*H*)-quinolone	ER, ERB, ERO	Acute toxicity	Exhibited certain acute toxicity with the LD_50_ values of 64.9 mg/kg in Kunming mice	[50]
			MAO-B inhibitors	Showed a selective inhibition of MAO-B activity with the IC_50_ of 15.3 μM	[51]
			Antitumor activity	Inhibited proliferation of HL-60, N-87, H-460, CCRF-CEM and Hep G2 cells with IC_50_ values of 21.64, 20.52, 21.08, 4.56 and 19.75 μM, respectively; showed cytotoxic activities against PC-3 with GI_50_ of 17.61 μM	[20, 47, 52]
			P-gp modulators	Showed cytotoxic activities against p-gp over-expressing subline CEM/ADR5000 with IC_50_ value of 17.19 μM	[52]
			Antibacterial activity	Against *Staphyloccocus aureus* ATCC25923, *Staphylococcus epidermidis* ATCC12228 with MIC values of 64 and 32 μg/mL, respectively	[43]
63	1-Methyl-2-[(*Z*)-1-undecenyl]-4(1*H*)-quinolone	ER	Antitumor activity	Had moderate cytotoxicity against tumor cell lines Lovo, MDA-MB-231 and HeLa with IC_50_ values of 6.72, 14.20 and 13.05 μM, respectively	[53]
64	1-Methyl-2-[(*E*)-1-undecenyl]-4(1*H*)-quinolone	ER	Antitumor activities	Inhibited proliferation of human tumor HL-60, N-87, H-460, and Hep G2 cells with IC_50_ of 18.36, 18.04, 20.11 and 21.91 μM, respectively	[47]
65	1-Methyl-2-[(*Z*)-5-undecenyl]-4(1*H*)-quinolone	ER	Antitumor activities	Showed cytotoxic activities against HL60 with GI_50_ of 54.10 ± 0.27 μM	[20]
			Antibacterial activity	Against methicillin-resistant *Staphylococcus aureus* (MRSA) with MIC value of 32 μg/mL	[49]
66	1-Methyl-2-[(*Z*)-6-undecenyl]-4(1*H*)-quinolone	ER	Leukotriene biosynthesis inhibitors	Inhibited leukotriene biosynthesis in a bioassay using human polymorphonuclear granulocytes with IC_50_ of 10.0 μM	[46]
			Antitumor activities	Inhibited proliferation of HL-60, N-87, H-460, and Hep G2 cells with IC_50_ of 18.80, 18.66, 20.00 and 19.45 μM, respectively; showed cytotoxic activity against both A549 and HT-29 cells with IC_50_ of 2.2 and 5.3 μg/mL	[20, 47, 52]
			MAO-B inhibitor	Inhibited MAO-B activity with IC_50_ values of 13.5 μM	[48]
67	1-Methyl-2-[(1*E*,5*Z*)-1,5-undecadienyl]-4(1H)-quinolone	ER	Antitumor activities	Inhibited proliferation of human tumor HL-60, N-87, H-460, and Hep G2 cells with IC_50_ of 19.56, 16.70, 19.97 and 16.41 μM, respectively	[47]
68	1-Methyl-2-[6-carbonyl-(*E*)-4-undecenyl]-4(1H)-quinolone	ER	Antitumor activities	Showed cytotoxic activities against HL60 with GI_50_ of 58.13 ± 1.45 μM	[20]
69	1-Methyl-2-undecanone-10'-4(1*H*)-quinolone	ERB	Acute toxicity	Exhibited certain acute toxicity with the LD_50_ values of 47.6 mg/kg in vivo	[50]
70	1-Methyl-2-dodecyl-4-(1*H*)-quinolone	ER, ERO	Antitumor activities	Inhibited proliferation of human tumor HL-60, N-87, H-460, and Hep G2 cells with IC_50_ of 17.66, 20.82, 18.99 and 16.52 μM, respectively	[47]
			Antibacterial activity	Against *Staphyloccocus aureus* ATCC25923, *Staphylococcus epidermidis* ATCC12228 and *Bacillus subtilis* ATCC6633 with MIC values of 64, 16 and 64 μg/mL, respectively	[43]
71	1-Methyl-2-[(*Z*)-5'-dodecenyl]-4(1*H*)- quinolone	ER			[43]
72	Dihydroevocarpine	ER, ERO	NFAT inhibitor	Showed inhibitory effects against NFAT activity with IC_50_ of 5.48 ± 0.30 μM	[45]
			Antitumor activities	inhibited proliferation of HL-60, N-87, H-460, CCRF-CEM and Hep G2 cells with IC_50_ of 15.41, 18.18, 16.53, 14.08 and 19.90 μM, respectively; Showed cytotoxic activity against A549, HT-29 and Hela cells with IC_50_ of 11.5, 23.9 and 26.98 μM, respectively	[20, 43, 47, 52, 54]
			P-gp modulators	Showed cytotoxic activities against p-gp over-expressing subline CEM/ADR5000 with IC_50_ value of 33.14 μM	[52]
			Antibacterial activity	Against *Staphyloccocus aureus* ATCC25923, *Staphylococcus epidermidis* ATCC12228, and *Bacillus subtilis* ATCC6633 with MIC values of 4, 4 and 8 μg/mL, respectively	[43]
73	Evocarpine	ER, ERO	Anti-inflammatory activities	Inhibited fMLP/CB-induced elastase release with IC_50_ values of 2.6 ± 0.26 μM	[19]
			NFAT inhibitor	Showed inhibitory effects against NFAT inhibitor activity with IC_50_ of 1.09 μM	[45]
			Leukotriene biosynthesis inhibitors	Inhibited the leukotriene biosynthesis in a bioassay with IC_50_ values of 14.6 μM	[46]
			Antitumor activities	Inhibited proliferation of HL-60, N-87, H-460, Hep G2, A549 and HT-29 cells with IC_50_ of 18.12, 17.25, 17.34, 20.38, 5.4 and 8.2 μM, respectively; Showed cytotoxic activities against PC-3 with GI_50_ of 15.11 μM	[20, 47, 52]
			DGAT inhibitors	Showed a dose-dependent DGAT inhibition with IC_50_ values of 23.8 μM	[55]
			Antibacterial activity	Against MRSA with MIC value of 8 μg/mL; against *Staphyloccocus aureus* ATCC25923, *Staphylococcus epidermidis* ATCC12228, and *Bacillus subtilis* ATCC6633 with MIC values of 64, 16 and 32 μg/mL, respectively	[43, 49]
74	Euocarpine A	ER	Antibacterial activity	Against *Staphyloccocus aureus* ATCC25923 and *Staphylococcus epidermidis* ATCC12228 MIC value of 128 μg/mL	[43]
75	Euocarpine B	ER	Antibacterial activity	Against *Staphyloccocus aureus* ATCC25923, and *Staphylococcus epidermidis* ATCC12228 with MIC values of 128 and 128 μg/mL, respectively	[43]
			Antitumor activities	Exhibited cytotoxic activities against HepG-2, Hela, BEL7402, and BEL7403 with IC_50_ of 52.8, 24.25, 24.57 and 33.54 μM, respectively	
76	Euocarpine C	ER	Antibacterial activity	Against *Staphyloccocus aureus* ATCC25923 with MIC value of 64 μg/mL	[43]
77	Euocarpine D	ER	Antitumor activities	Showed cytotoxic activities against HL60 with GI_50_ of 73.14 ± 0.71 μM	[20]
			Antibacterial activity	Against *Staphyloccocus aureus* ATCC25923, *Staphylococcus epidermidis* ATCC12228, and *Bacillus subtilis* ATCC6633 with MIC values of 32, 16 and 16* μg*/mL, respectively	[43]
78	Euocarpine E	ER	Antibacterial activity	Against *Staphyloccocus aureus* ATCC25923, *Staphylococcus epidermidis* ATCC12228, and *Bacillus subtilis* ATCC6633 with MIC values of 64, 32 and 32 μg/mL, respectively	[43]
79	1-Methyl-2-[(*Z*)-4-tridecenyl]-4(1*H*)-quinolone	ER			[16]
80	1-Methyl-2-[(*Z*)-7-tridecenyl]-4(1*H*)- quinolone	ER	Antitumor activities	Showed cytotoxic activities against HL60 with GI_50_ of 21.04 ± 0.50 μM	[20]
			Antibacterial activity	Showed inhibitory against *Helicohacter pylori* strains and MIC was less than 0.05 μg/mL	[56]
81	1-Methyl-2-[(*Z*)-8-tridecenyl]-4(1*H*)-quinolone	ER	Antitumor activity	Had cytotoxicity against Lovo, MDA-MB-231 and HeLa cells with IC_50_ values of 20.78, 15.85 and 15.77 μM, respectively	[53]
			Antibacterial activity	Showed inhibitory against *Helicohacter pylori* strains and MIC was less than 0.05 μg/mL	[56]
82	1-Methyl-2-[12-tridecenyl]-4(1*H*)-quinolone	ER			[57]
83	1-Methyl-2-[(4*Z*,7*Z*)-4,7-tridecadienyl]-4(1*H*)-quinolone	ER, ERO	NFAT and NF-кB inhibitors	Inhibited NFAT and NF-кB activity with respective IC_50_ values of 1.86 and 10.80 μM	[45]
			Leukotriene biosynthesis inhibitors	Exhibited inhibitory activity on leukotriene biosynthesis in a bioassay with IC_50_ of 10.1 μM	[46]
			Antitumor activity	Had moderate cytotoxicity against Lovo, MDA-MB-231 and HeLa cells with IC_50_ values of 18.17, 8.25 and 13.05 μM, respectively; inhibited proliferation of HL-60, N-87, H-460, and Hep G2 cells with IC_50_ values of 18.50, 17.85, 16.03 and 19.83 μM, respectively	[20, 47, 53]
			DGAT inhibitors	Showed a dose-dependent DGAT inhibition with IC_50_ values of 20.1 μM	[55]
84	1-Methyl-2-[6-carbonyl-(*E*)-7-tridecenyl]-4(1*H*)-quinolone	ER			[20]
85	1-Methyl-2-[7-carbonyl-(*E*)-9-tridecenyl]-4(1*H*)-quinolone	ER	Antitumor activities	Showed cytotoxic activities against HL60 with GI_50_ of 30.84 ± 2.62 μM	[20]
86	1-Methyl-2-[7-hydroxy-(*E*)-9-tridecenyl]-4(1*H*)-quinolone	ER	Antitumor activities	Inhibited proliferation of HL-60, N-87, H-460 and Hep G2 cells with IC_50_ values of 18.26, 16.25, 13.27 and 14.36 μM, respectively	[47]
87	1-Methyl-2-[12-hydroxy-tridecyl]-4(1H)-quinolone	ER			[57]
88	1-Methyl-2-[13-hydroxyl-tridecenyl]-4(1*H*)-quinolone	ER	Antitumor activities	Showed cytotoxic activities against HL60 with GI_50_ value of 12.07 ± 2.28 μM	[20]
89	1-Methyl-2-tetradecyl-4-(1*H*)-quinolone	ER	Antitumor activities	Inhibited proliferation of HL-60, N-87, H-460 and Hep G2 cells with IC_50_ values of 17.72, 16.72, 15.54 and 16.83 μM, respectively	[47]
			Antibacterial activity	Against *Staphyloccocus aureus* ATCC25923 and *Staphylococcus epidermidis* ATCC12228, with MIC values of 16 and 4 μg/mL, respectively	[43]
90	1-Methyl-2-[13-tetradecenyl]-4-(1*H*)-quinolone	ER			[57]
91	1-Methyl-2-pentadecyl-4(1*H*)-quinolone	ER, ERO	NFAT inhibitor	Inhibited NFAT activity with IC_50_ values of 0.91 μM	[45]
			Antitumor activities	Inhibited proliferation of HL-60, N-87, H-460 and Hep G2 cells with IC_50_ values of 17.54, 14.27, 15.79 and 15.95 μM, respectively; exhibited moderate cytotoxic activities against Hela, BEL7402 and BEL7403 cells with IC_50_ of 23.36, 29.51 and 36.86 μM, respectively	[43, 47]
			Antibacterial activity	Against *Staphyloccocus aureus* ATCC25923, *Staphylococcus epidermidis* ATCC12228 with MIC values of 16 and 4 μg/mL, respectively	[43]
92	1-Methyl-2-[(*Z*)-5'-pentadecenyl]-4(1*H*)-quinolone	ER	Antibacterial activity	Against *Staphyloccocus aureus* ATCC25923, *Staphylococcus epidermidis* ATCC12228, and *Bacillus subtilis* ATCC6633 with MIC values of 16, 4 and 16 μg/mL, respectively	[43]
			Antitumor activities	Exhibited cytotoxic activities against HepG-2, Hela, BEL7402 and BEL7403 with IC_50_ of 49.83, 18.53, 15.85 and 35.83 μM, respectively	[43]
93	1-Methyl-2-[(*Z*)-6-pentadecenyl]-4(1H)-quinolone	ER			[47]
94	1-Methyl-2-[(*Z*)-9-pentadecenyl]-4(1H)-quinolone	ER, ERO			[58]
95	1-Methyl-2-[(*Z*)-10-pentadecenyl]-4(1H)-quinolone	ER, ERO			[47]
96	1-Methyl-2-[(6*Z*,9*Z*)-6,9-pentadecadienyl]-4(1*H*)-quinolone	ER, ERO	NFAT and NF-κB inhibitors	Showed inhibitory effects against NFAT and NF-кB with IC_50_ values of 1.01 and 6.60 μM, respectively	[45]
			Leukotriene biosynthesis inhibitors	Inhibited the leukotriene biosynthesis in a bioassay using human polymorphonuclear granulocytes with IC_50_ values of 12.3 μM	[46]
			Antitumor activities	Inhibited proliferation of HL-60, N-87, H-460 and Hep G2 cells with IC_50_ of 16.1, 12.6, 16.7 and 15.3 μM, respectively	[47]
			DGAT inhibitors	Showed a dose-dependent DGAT inhibition with IC_50_ values of 13.5 μM	[55]
			MAO-B inhibitor	Inhibited MAO activity dose-dependently with IC_50_ values of 3.6 μM	[48]
			Antibacterial activity	Against methicillin-resistant *Staphylococcus aureus* with MIC value of 128 μg/mL	[49]
97	1-Methyl-2-[(9*E*,13*E*)-eptadecadienyl]-4 (1*H*)-quinolone	ER			[57]
98	1-Methyl-2-[(6*Z*,9*Z*,12*Z*)-6,9,12-pentadecatriene]-4(1*H*)-quinolone	ER	Antitumor activities	Exhibited potent activity against MDA-MB-231 cells with IC_50_ values of 7.95 μM	[59]
99	1-Methyl-2-[(6*Z*,9*Z*,12*E*)-pentadecatriene]-4 (1*H*)-quinolone	ER			[60]
100	1-Methy-l-2-[15-hydroxyl-pentadecenyl]-4(1*H*)-quinolone	ER	Antitumor activities	Showed cytotoxic activities against both HL60 and PC-3 with GI_50_ of 20.36 and 31.99 μM	[20]
101	1-Methyl-2-hexadecylol-4-(1*H*)-quinolone	ER			[57]
102	2-Nonyl-4(1*H*)-quinolone	ER	NFAT inhibitor	Showed inhibitory against NFAT activity with IC_50_ values of 3.44 ± 0.04 μM	[45]
103	2-Undecyl-4(1*H*)-quinolone	ER, ERB	Acute toxicity	Exhibited acute toxicity with the LD_50_ values of 36.1 mg/kg in Kunming mice	[50]
			NFAT inhibitor	Showed inhibitory against NFAT activity with IC_50_ values of 3.29 ± 0.02 μM	[45]
104	2-Undecanone-10'-4(1H)-quinolone	ERB			[50]
105	2-Tridecyl- 4(1H)-quinolone	ER			[58]
106	2-[(6*Z*,9*Z*)-Pentadeca-6,9-dienyl]-quinolin-4(1*H*)-one	ER			[19]
107	Atanine	ER	Antitumor activity	Exhibited cytotoxicity against Jurkat and RAJI cell with IC_50_ of 14.5 and 9.3 μg/mL, respectively	[61]
108	2-Hydroxy-4-methoxy-3(3'-methyl-2'-butenyl)-quinolin	ERO	Antitumor activity	Showed cytotoxicity against A549 and HT-29 cells with respective IC_50_ of 9.9 and 12.0 μg/mL	[54]
109	3-(3-Hydroxy-3-methylbutyl)-4-methoxyquinolin-2(1 *H*)-one	ER			[62]
110	4-Hydroxy-3-(3-hydroxy-3-methylbutyl)-quinolin-2(1*H*)-one	ER			[62]
111	Quinolone A	ER			[63]
112	Quinolone B	ER			[63]
113	Evodiamide A	ER			[29]
114	Evodiamide B	ER			[29]
115	Evodiamide C	ER			[29]
116	Evodiaxinine	ER			[29]
117	Skimmianine	ER	Anti-inflammatory activity	Inhibited fMLP/CB-induced O_2_˙ˉ generation and elastase release with IC_50_ values of 20.9 ± 3.5 and 14.4 ± 1.3 μM, respectively	[19]
118	Dictamnine	ER			[41]
119	Evolitrine	ER			[41]
120	6-Methoxydictamnine	ER			[41]
121	Evodine	ER			[64]
122	Ribalinine	ER			[60]
123	8-Hydroxy-4-methoxy-3-(3-methylbut-2-en-1-yl) quinolin-2(1*H*)-one	ER			[22]
124	(*S*)-3-(2-Hydroxy-3-methylbut-3-en-1-yl)-4-methoxyquinolin-2(1*H*)-one	ER			[22]
125	Limonelone	ER			[22]
126	2-Methyl-4(3*H*)-quinazolinone	ER			[22]
127	Synephrine	ER	Vasoconstrictive activity	Showed constrictive effects on rat aorta at concentration of 1 × 10^−7^–3 × 10^−5^ mol/L	[65]
128	*N*-(*trans*-*p*-Coumaroyl)-tyramine	ER			[41]
129	*N*-(*cis*-*p*-Coumaroyl)-tyramine	ER			[41]
130	*N*-Methylanthranylamide	ER			[38]
131	Berberine	ER			[66]
132	Salsoline A	ER			[67]
133	Caffeine	ER			[68]
Terpenoids					
134	Limonin	ER, ERB	Anti-inflammatory activity	Significantly inhibited the AA-induced ear edema at a dose of 100 mg/kg	[69]
			Insecticidal activity	Against Asian tiger mosquitoes with LC_50_ values of 32.43 μM	[30, 31]
			Neuroprotective effect	Alleviated serum-deprivation induced P12 cell damage, increasing the cell viability from 55.5 ± 5.0 to 83.5 ± 5.3% at the concentration of 10 μM	[70]
135	12*α*-Hydroxylimonin	ER	Neuroprotective effect	Alleviated serum-deprivation induced P12 cell damage, increasing the cell viability from 55.5 ± 5.0 to 81.0 ± 3.7% at the concentration of 10 μM	[70]
136	Dehydrolimonin	ER			[71]
137	Limonin 17-*β*-D-glucopyranoside	ER			[72]
138	Rutaevin	ER, ERB	Anti-inflammatory activity	Inhibited NO production in lipopolysaccharide-activated RAW264.7 macrophages with 151.6 μM	[73]
139	Rutaevin acetate	ER			[18]
140	12*α*-Hydroxyrutaevin	ERB	Anti-inflammatory activity	Inhibited NO production in lipopolysaccharide-activated RAW264.7 macrophages with 161.5 ± 5.0 μM	[73]
141	Evodol	ER	Anti-inflammatory activity	Inhibited fMLP/CB-induced elastase release with IC_50_ values of 11.7 μM	[19]
			Insecticidal activity	Against *M. incognita* and Asian tiger mosquitoes with respective LC_50_ of 155.02 and 52.22 μg/mL	[30, 31]
142	12*α*-Hydroxyevodol	ER			[18]
143	6*α*-Acetoxyl-12*α*-hydroxyevodol	ER			[62]
144	Limonin diosphenol 17-*β*-D-glucopyranoside	ER			[72]
145	Jangomolide	ER			[18]
146	6*α*-Acetoxy-5-epilimonin	ER			[18]
147	6*β*-Acetoxy-5-epilimonin	ER			[18]
148	6*β*-Hydroxy-5-epilimonin-17*β*-D-glucopyranoside	ER			[72]
149	Evorubodinin	ERB	Anti-inflammatory activity	Inhibited NO production in lipopolysaccharide-activated RAW264.7 macrophages with IC_50_ value of 218.3 ± 3.3 μM	[73]
150	Shihulimonin A	ERB	Anti-inflammatory activity	Inhibited NO production in lipopolysaccharide-activated RAW264.7 macrophages with IC_50_ value of 180.2 ± 9.5 μM	[73]
151	Evolimorutanin	ERB	Anti-inflammatory activity	Inhibited NO production in lipopolysaccharide-activated RAW264.7 macrophages with IC_50_ value of 182.9 ± 4.1* μM*	[73]
152	Evodirutaenin	ERB	Anti-inflammatory activity	Inhibited NO production in lipopolysaccharide-activated RAW264.7 macrophages with IC_50_ value of 246.9 ± 7.8 μM	[73]
153	Isolimonexic acid	ER			[53]
154	Obacunonsaeure	ER			[62]
155	Obacunone	ER			[18]
156	7-Deacetylproceranone	ER			[70]
157	Nomilin	ER	Neuroprotective effect	Alleviated serum-deprivation induced P12 cell damage, increasing the cell viability from 55.5 to 88.6% at 10 μM	[70]
158	Isoobacunoic acid	ER			[62]
159	12-Ursen-3-ol	ER			[74]
160	14-Ursen-3-ol-1-one	ER			[68]
161	Glycyrrhetinic	ER			[75]
162	Glycyrrhizic acid	ER			[75]
163	Taraxerone	ER			[68]
164	Oleanolic acid	ER			[66]
165	Evoditrilone A	ER	Antitumor activity	Showed antitumor activity against A549 and LoVo cells with IC_50_ values of 2.0 and 1.9 μM, respectively	[70]
166	Evoditrilone B	ER	Neuroprotective effect	Alleviated serum-deprivation induced P12 cell damage, increasing the cell viability from 55.5 ± 5.0 to 80.3 ± 6.1% at the concentration of 10 μM	[70]
167	Ursolic acid	ER			[70]
168	3*β*-Hydroxyoleana-11,13(18)-diene	ER			[70]
169	1*β*, 4*β*-Dihydroxyeudesman-11-ene	ER			[67]
Steroids					
170	*β*-Sitosterol	ER, ERO			[19]
171	Stigmasterol	ER, ERO			[19]
172	3*β*-Hydroxystigmast-5-en-7-one	ER			[19]
173	3*β*-Hydroxystigmasta-5,22-dien-7-one	ER			[19]
174	Daucosterol	ER, ERO			[62]
Phenols					
175	Tricin-7-*O*-*β*-D-glucopyranoside	ER			[67]
176	Diosmetin-7-*O*-*β*-D-glucopyranoside	ER			[76]
177	Diosmin	ER			[76]
178	Chrysoeriol-7-*O*-rutinoside	ER			[76]
179	Isorhamnetin	ER			[76]
180	Isorhamnetin 3-*O*-*β*-D-galactoside	ER			[25]
181	Isorhamnetin-3-*O*-*β*-D-glucopyranoside	ER			[77]
182	Isorhamnetin 3-*O*-rutinoside	ER, ERO			[78]
183	Isorhamnetin-3-*O*-*β*-D-xylopyranosyl(1 → 2)-*β*-D-glucopyranoside	ER			[77]
184	Isorhamnetin-3-*O*[2-*O*-*β*-D-xylopyranosyl-6-*O*-*α*-L-rhamnopyranosyl]-*β*-D-glucopyranoside	ER			[77]
185	Quercetin	ER			[67]
186	Isoquercitrin	ER			[77]
187	Quercetin 3-*O*-*β*-D-galactoside	ER			[79]
188	Quercetin 3-*O*-*β*-D-xylopyranosyl (1 → 2)-*β*-D-glucopyranoside	ER			[77]
189	Limocitrin3-*O*-*β*-D-glucopyranoside	ER			[77]
190	Limocitrin 3-*O*-rutinoside	ER, ERO	DNA topoisomerase inhibitor	Showed strong inhibitory effects on DNA topoisomerases I and II (70 and 96% inhibition at a concentration of 20 μM, respectively)	[78]
191	Limocitrin 3-*O*-*β*-D-xylopyranosyl (1 → 2)-*β*-D-glucopyranoside	ER			[77]
192	Limocitrin3-*O*[2-*O*-*β*-D-xylopyranosyl-6-*O*-*α*-L-rhamnopyranosyl]-*β*-D-glucopyranoside	ER			[77]
193	Hyperoside	ER			[60]
194	Veronicafolin3-rhamnosyl-glucoside	ERO			[78]
195	Phellodensin F	ER			[67]
196	Epimedoside C	ER			[80]
197	Flavaprin	ER			[67]
198	Evodioside B	ER			[16]
199	Hesperidin	ER			[81]
200	Catechin	ER			[67]
201	Cinchonain	ER			[67]
202	Chrysophanol	ERO			[82]
203	Emodin	ERO			[82]
204	Physcion	ERO			[82]
205	Neochlorogenic acid	ER			[83]
206	Chlorogenic acid	ER			[60]
207	3-*O*-Caffeoylquinic acid methyl ester	ER			[84]
208	Caffeic acid	ER			[81]
209	*trans*-Caffeic acid methylate	ER			[57]
210	Ferulic acid	ER			[84]
211	p-Hydroxycinnamic acid	ER			[84]
212	Methyl coumarate	ER			[68]
213	2-*O*-*trans*-Caffeoylgluconic acid	ER	Hepatotoxicity	The mixture had certain toxicity to L02 cells with IC_50_ values of 319.8 μM at 12 h	[83]
214	3-*O*-*trans*-Caffeoylgluconic acid				[83]
215	4-*O*-*trans*-Caffeoylgluconic acid				[83]
216	5-*O*-*trans*-Caffeoylgluconic acid	ER			[83]
217	6-*O*-*trans*-Caffeoylgluconic acid	ER			[83]
218	trans-Caffeoyl-6-*O*-D-gluconic acid methyl ester	ER			[83]
219	trans-Caffeoyl-6-*O*-D-glucono-*γ*-lactone	ER			[83]
220	*trans*-Feruloylgluconic acid	ER			[57]
221	9-*O*-Feruloyl-4-*O*-*β*-D-glucopyanoside	ER			[84]
222	p-Hydroxybenzoic acid ethyl ester	ER			[66]
223	Isovanillin	ER			[74]
224	3,4-Dihydroxy-benzoic acid	ER			[84]
225	7-Hydroxy coumarin	ER			[84]
Others					
226	Ruticarpside A	ER			[85]
227	Ruticarpside B	ER			[85]
228	Ruticarpside C	ER			[85]
229	Evodinoid A	ER			[86]
230	Evodinoid B	ER			[86]
231	Syringoside	ER			[71]
232	Coniferin	ER			[60]
233	Citric acid	ER			[74]
234	4-Methoxybenzylalcohol	ER			[84]
235	*myo*-Inositol	ER			[87]
236	Phthalic acid dibutyl ester	ER			[87]
237	2-Pentadecanone	ER			[74]
238	1-Octadecanol	ER			[74]
239	Glycerol 1-octadecanoate	ER			[74]
240	n-Heptacosanol	ER			[74]

ER: *Euodia rutaecarpa* (Juss.) Benth.; ERO: *E. rutaecarpa* (Juss.) Benth. var. *officinalis* (Dode) Huang; ERB: *E. rutaecarpa* (Juss.) Benth. var. *bodinieri* (Dode) Huang

### Alkaloids

The alkaloids extracted from Euodiae Fructus have attracted wide attention from chemists and pharmacologists due to their various biological effects. Among these compounds, indole alkaloids and quinolone alkaloids are the main structural types.

**Fig. 2:**
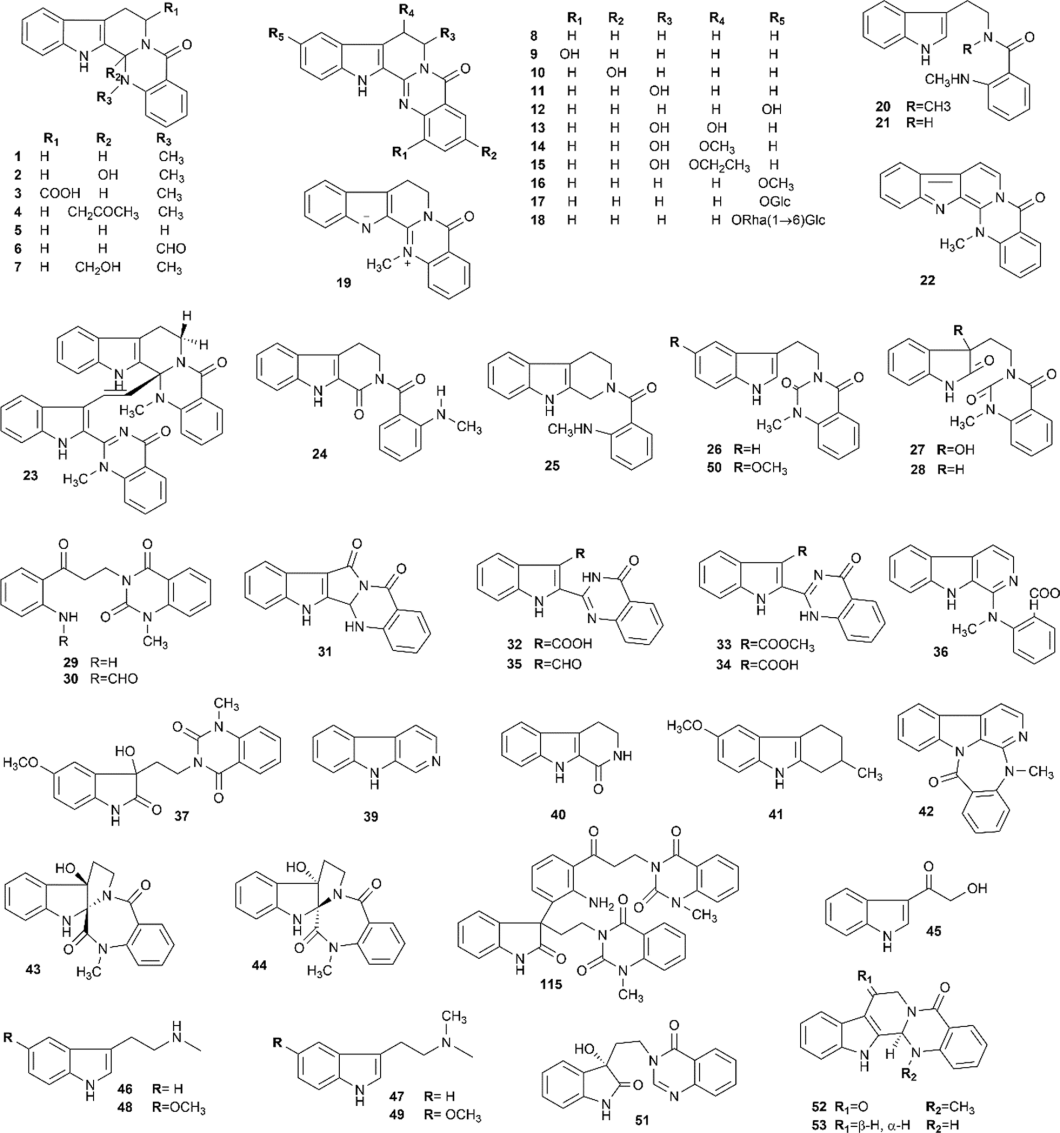
Indole alkaloids isolated from Euodiae Fructus

Up to 53 indole alkaloids were isolated from Euodiae Fructus, and their structures are shown in Fig. 2. Evodiamine, rutaecarpine and dehydroevodiamine are regarded as the dominant chemical constituents with a wide range of pharmacological activities.

**Fig. 3:**
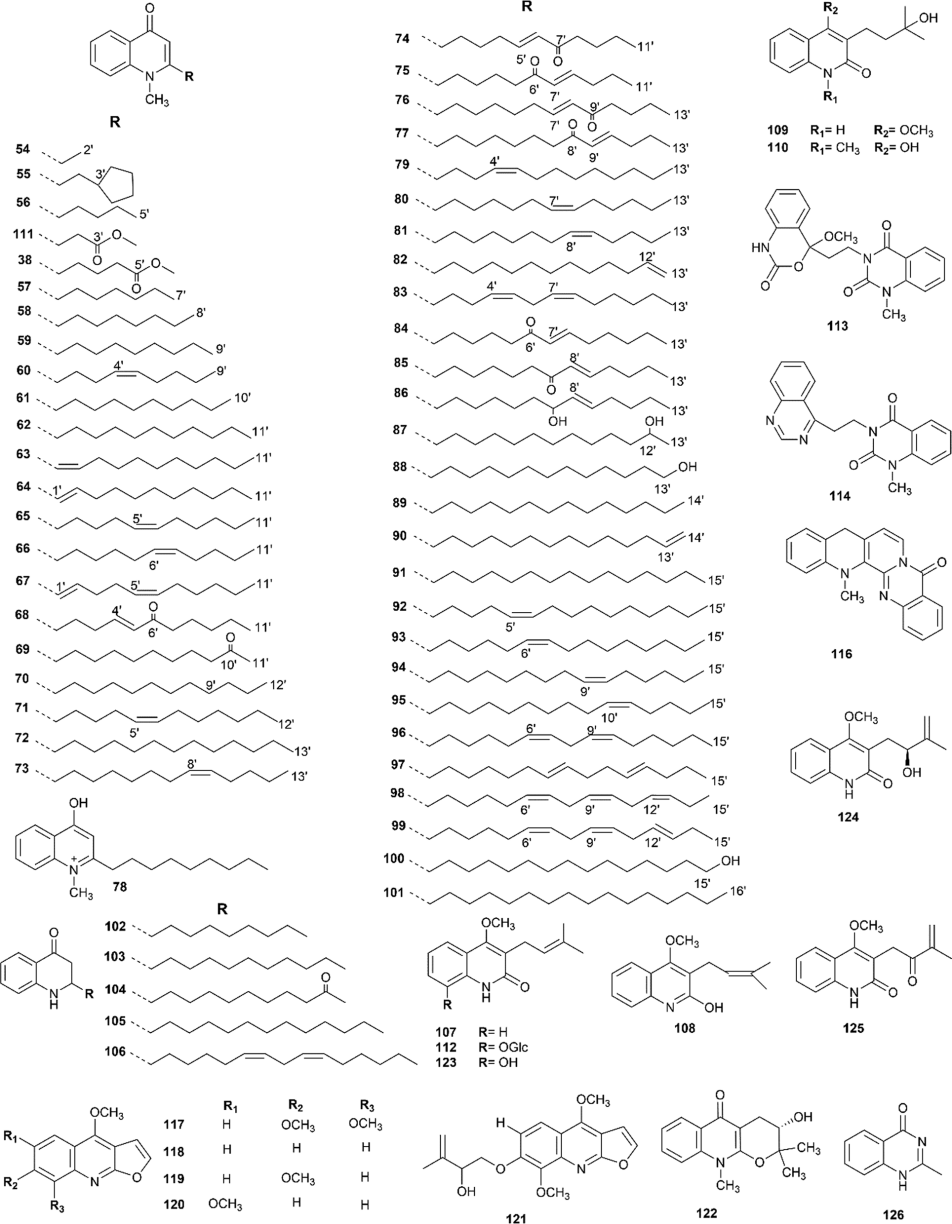
Quinoline alkaloids isolated from Euodiae Fructus

There are 73 quinolone alkaloids extracted from Euodiae Fructus and their structure are shown in Fig. 3. Among them, quinolinone with an alpha-substituted saturated or unsaturated aliphatic hydrocarbon group is the typical structures of these compounds [6]. 1-Methyl-2-undecyl-4(1*H*)-quinolone is a representative constituent of these compounds, which has been reported to exhibit anticancer activity [20, 47], anti-calcific aortic stenosis [88], and monoamine oxidase type B (MAO-B) inhibitory [51].

**Fig. 4:**
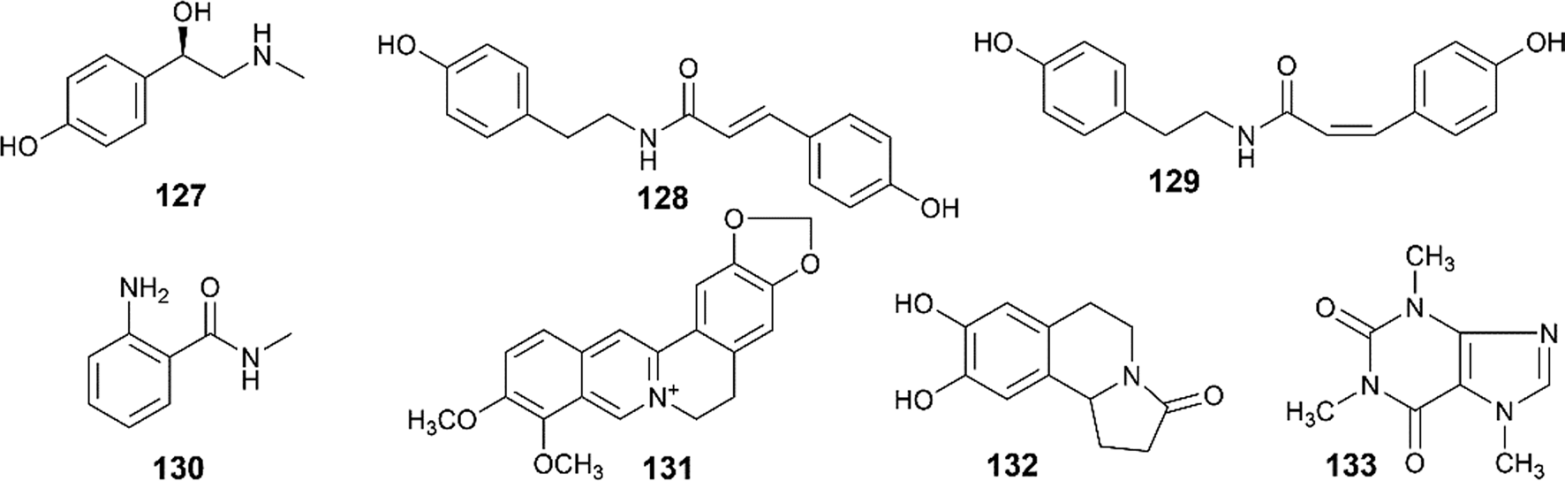
Other alkaloids isolated from Euodiae Fructus

Other types of alkaloids have also been isolated from plants of Euodiae Fructus, including berberine, synephrine, caffeine, *N*-methylanthranylamide, *N*-(*trans*-*p*-coumaroyl)-tyramine, *N*-(*cis*-*p*-coumaroyl)-tyramine, etc. Their structures are shown in Fig. 4.

### Terpenoids

**Fig. 5:**
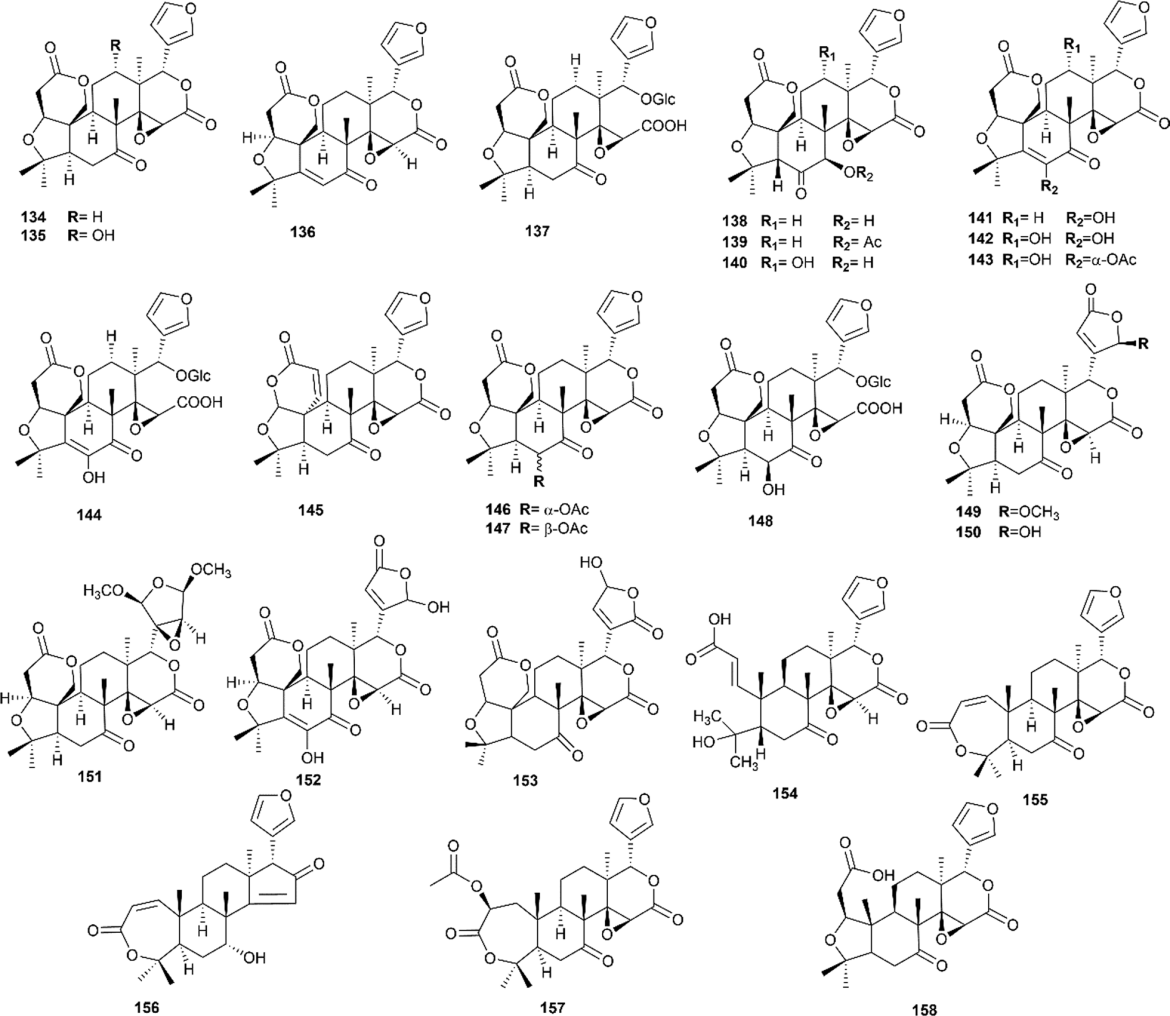
Limonoids isolated from Euodiae Fructus

**Fig. 6:**
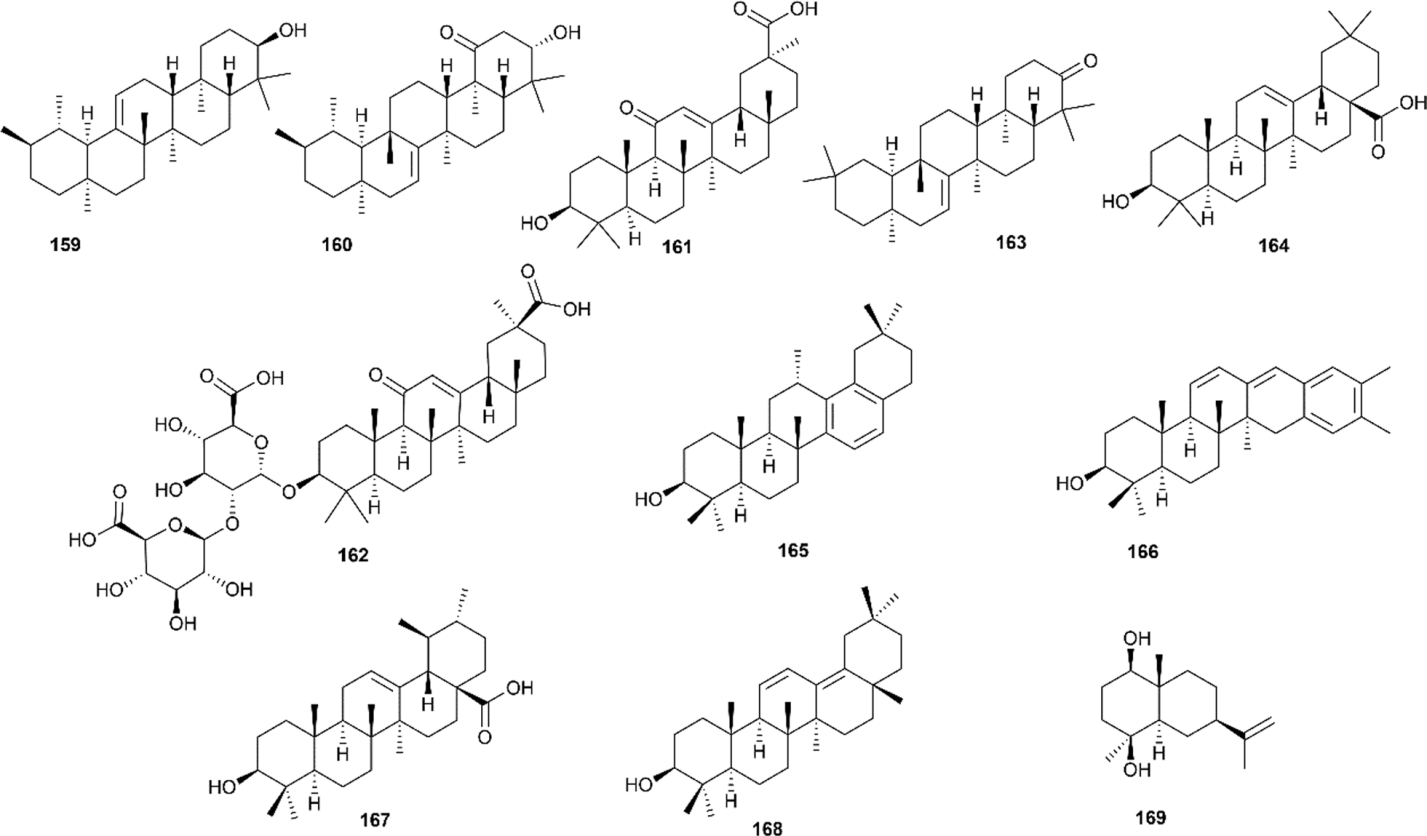
Other terpenoids isolated from Euodiae Fructus

There are 36 terpenoids (25 limonoids, 10 triterpenoids and a diterpenoid) also isolated from this plant, and their structures are presented in Figs. 5, 6. Limonoids are highly oxidized tetracyclic triterpenoids with furan ring, in which limonin is the most typical bioactive limonoids from Euodiae Fructus. In 1988, Tohru et al. isolated seven known limonoids, including limonin (**134**), rutaevin (**138**), rutaevin acetate (**139**), graucin A (**140**), evodol (**141**), jangomolide (**145**), obacunone (**155**), together with four new limonoids, 12*α*-hydroxylimonin (**135**), 12*α*-hydroxyevodol (**142**), 6*α*-acetoxy-5-epilimonin (**146**), 6*β*-acetoxy-5-epilimonin (**147**) [18]. In 1991, three limonoid glucosides, including limonin 17-*β*-D-glucopyranoside (**137**), limonin diosphenol 17-*β*-D-glucopyranoside (**144**) and 6*β*-hydroxy-5-epilimonin 17-*β*-D-glucopyranoside (**148**), were isolated from this plant [72]. In recent years, three new limonoids, such as evorubodinin (**149**), shihulimonin A (**150**) [73], and 6*α*-acetoxyl-12*α*-hydroxyevodol (**143**) [62], were first found from Euodiae Fructus, together with 12 known limonoids. Lately, an investigation of the 95% ethanol extract of Euodiae Fructus yielded two known limonoids (7-deacetylproceranone (**156**) and nomilin (**157**)), two novel nortriterpenoids (evoditrilones A (**165**) and B (**166**)), and three known triterpenoids (oleanic acid (**164**), ursolic acid (**167**), and 3*β*-hydroxyoleana-11,13(18)-diene (**168**)) [70]. Other triterpenoids mainly include 12-ursen-3-ol (**159**), 14-ursen-3-ol-1-one (**160**), glycyrrhizic acid (**161**), glycyrrhetinic (**162**) and taraxerone (**163**) [75].

### Steroids

**Fig.7:**
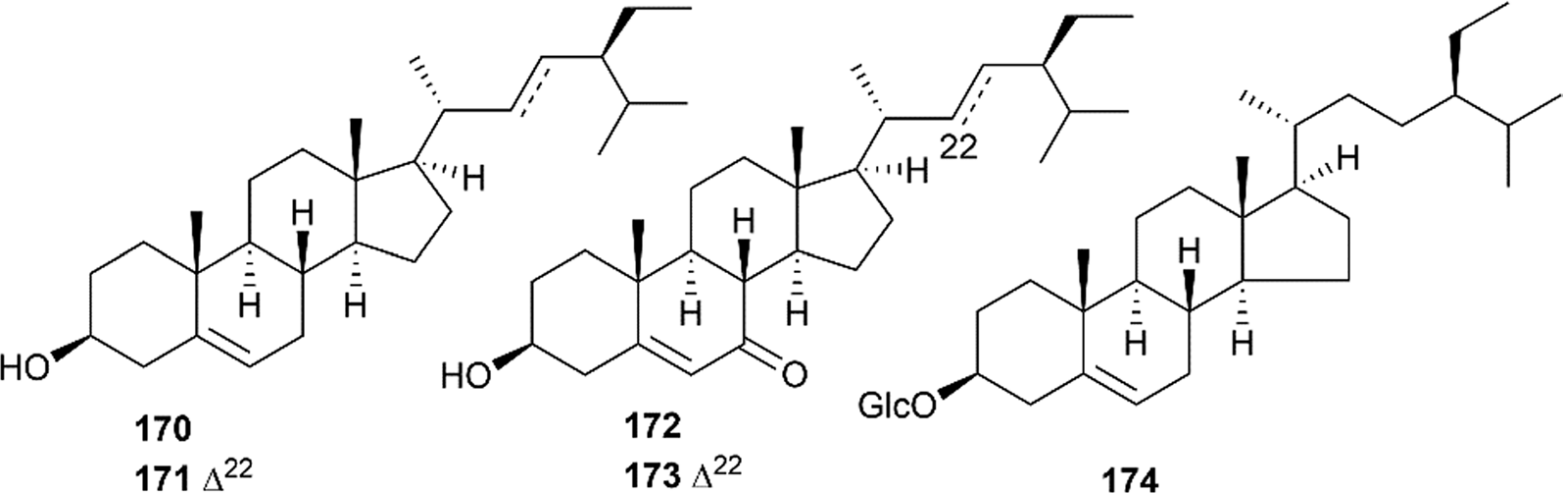
Steroids isolated from Euodiae Fructus

Phytosterols are a class of physiologically active constituents widely used in cosmetics, food and medicine. Steroids are relatively rare in Euodiae Fructus, and only five steroids were reported and characterized. In 2010, four steroids, namely, *β*-sitosterol (**170**), stigmasterol (**171**), *β*-hydroxystigmast-5-en-7-one (**172**) and 3*β*-hydroxystigmasta-5,22-dien-7-one (**173**), were found in methanol extract of the fruits of Euodiae Fructus [19]. In further studies, another steroid named daucosterol (**174**) was obtained from the 95% ethanol extract of Euodiae Fructus [81]. Their structures are presented in Fig. 7.

### Phenols

**Fig. 8:**
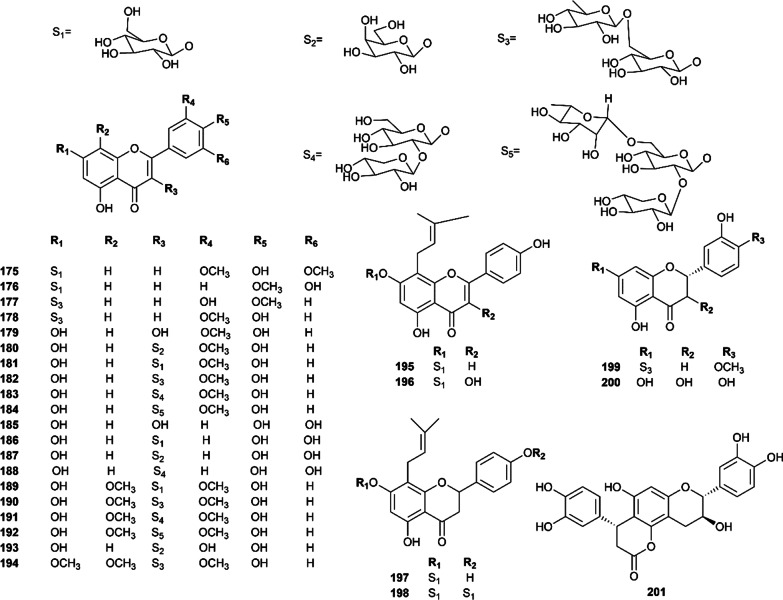
Flavonoids isolated from Euodiae Fructus

**Fig.9:**
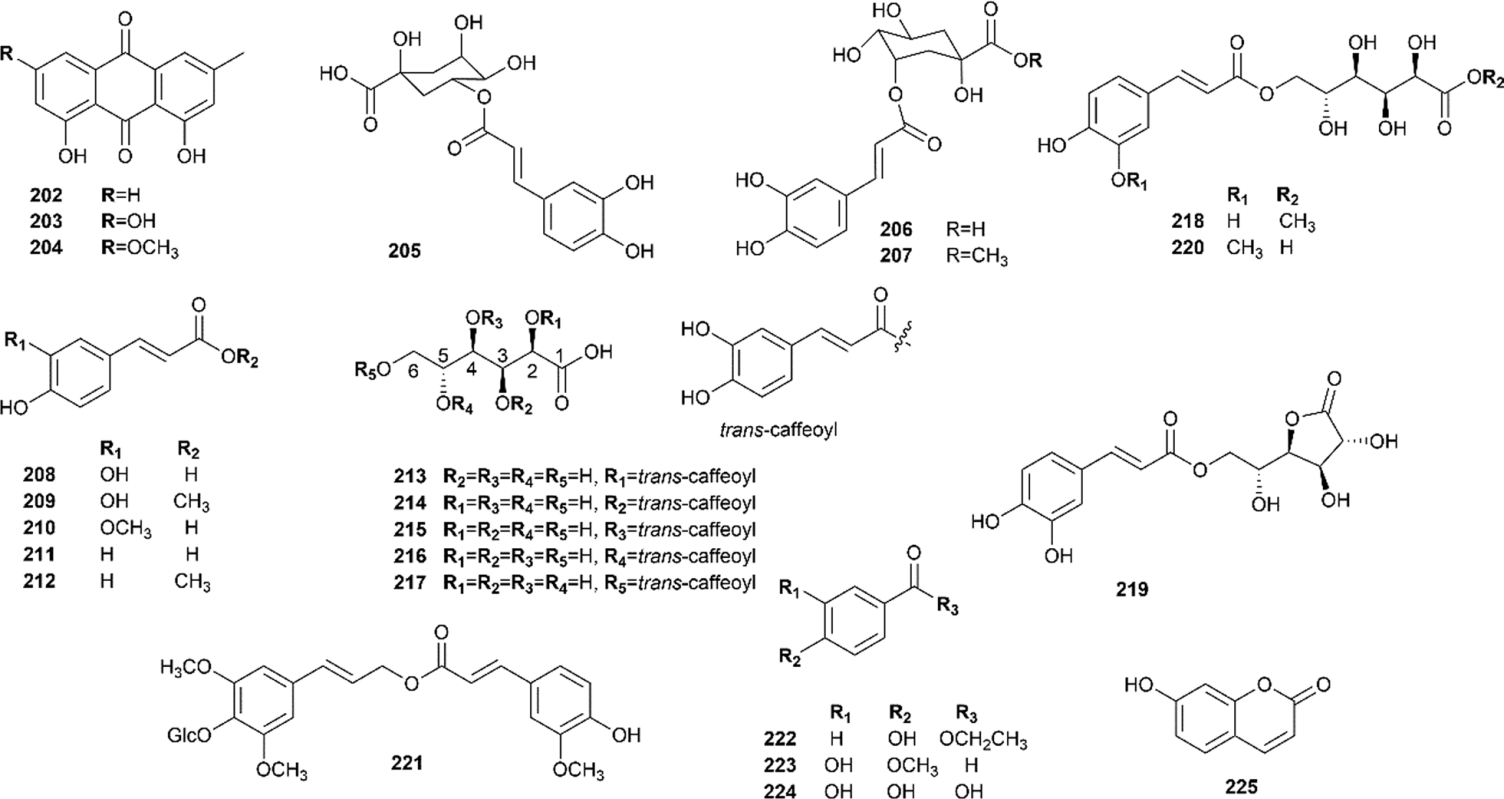
Other phenols isolated from Euodiae Fructus

To date, 51 phenols are characterized in this plant (Figs. 8, 9). Among them, 27 flavonoids were classified into three structural types, including flavones, tricin-7-*O*-*β*-D-glucopyranoside (**175**), diosmetin-7-*O*-*β*-D-glucopyranoside (**176**), diosmin (**177**), chrysoeriol-7-*O*-rutinoside (**178**) and phellodensin F (**195**) [67, 76]; flavonols, isorhamnetin, quercetin and their derivatives, which have been confirmed to exhibit a wide spectrum of pharmacological activities [89, 90]; and dihydroflavones, such as flavaprin (**197**), evodioside B (**198**) and hesperidin (**199**) [16, 67, 81].

Moreover, several phenolic acids and their derivatives have also been found in Euodiae Fructus. In 2013, caffeic acid (**208**) was isolated from the genus *Evodia* for the first time [81]. In recent years, He et al. isolated a new caffeoylgluconic acid derivative, *trans*-caffeoyl-6-*O*-d-gluconic acid methyl ester (**218**), together with two known compounds named *trans*-caffeoyl-6-*O*-d-gluconog-lactone (**219**) and *trans*-caffeoyl-6-*O*-D-gluconic acid (**217**) from Euodiae Fructus. Moreover, four new caffeoylgluconic acids, including 2-*O*-*trans*-caffeoylgluconic acid (**213**), 3-*O*-*trans*-caffeoyl-gluconic acid (**214**), 4-*O*-*trans*-caffeoylgluconic acid (**215**), 5-*O*-*trans*-caffeoylgluconic acid (**216**), together with three known ones including neochlorogenic acid (**205**), chlorogenic acid (**206**) and 3-*O*-caffeoylquinic acid methyl ester (**207**) were obtained from Euodiae Fructus [83]. In addition, *trans*-caffeic acid methylate (**209**), ferulic acid (**210**), *p*-hydroxycinnamic acid (**211**), *trans*-feruloylgluconic acid (**220**), *p*-hydroxybenzoic acid ethyl ester (**222**), 3,4-dihydroxy-benzoic acid (**224**) [84], and a new phenylpropanoid glycoside, 9-*O*-feruloyl-4-*O*-*β*-d-glucopyanoside (**221**) [84], were characterized in Euodiae Fructus. Additionally, chrysophanol (**202**), emodin (**203**), physcion (**204**) [82], and isovanillin (**223**), were successfully extracted from Euodiae Fructus.

### Volatile oil

The volatile oil is one of the main chemical compositions of Euodiae Fructus and its content is very high. Liu et al. identified 97 constituents by gas chromatography/mass spectrometer (GC/MS) analysis from 24 samples [91]. Another study showed that 97 constituents identified by SPME-GC–MS, accounted for 96.80% of volatile oil. Among the isolated volatile oil, the relative content of sesquiterpenes was more than 38%, monoterpenoids components was over 35%, ester components were above 13% [92]. It also indicated that the main constituents of the volatile oil from Euodiae Fructus were *β*-myrcene (17.7%), (*Z*)-*β*-ocimene (14.8%), *α*-phellandrene (14.7%), *γ*-terpinene (6.4%), linalool (5.7%) and *β*-thujene (5.1%) [93]. Moreover, several researches have been reported the volatile constituents obtained from Euodiae Fructus, such as caryophyllene oxide, linalool and *γ*-Elemene, have diverse functions, such as sedative, antiasthmatic, antibacterial, antitumor, antiviral and insect repellent, and its main components are caryophyllene oxide. It has been found that elemene is a new anticancer drug with great potential and has a broad clinical application prospect. Meanwhile, *γ*-Elemene can promote the immune function of erythrocytes [94]. However, modern toxicology studies showed that volatile oil could induce certain acute liver damage [95]. Taken together, the volatile oil may be efficacy material basis and toxicity material basis, but the research is isolated and lack of correlation, so further studies need be conducted to provide the experimental data and literature evidence for reasonable and safe development of the volatile oil from Evodia Fructus.

### Other compounds

**Fig.10:**
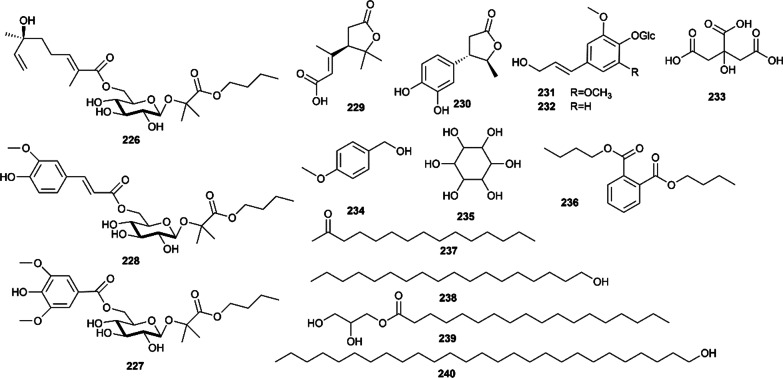
Other compounds isolated from Euodiae Fructus

Besides the above chemical constituents, syringoside (**231**), coniferin (**232**), citric acid (**233**), 4-methoxybenzylalcohol (**234**), *myo*-inositol (**235**), phthalic acid dibutyl ester (**236**) [87], and some fatty acids, such as 2-pentadecanone (**237**), 1-octadecanol (**238**), n-heptacosanol (**239**), glycerol 1-octadecanoate (**240**) [74]; three new ester glycosides, such as ruticarpside A (**226**), ruticarpside B (**227**) and ruticarpside C (**228**) [85], and two new *γ*-lactone derivatives, evodinoids A (**229**) and B (**230**) [86], have also been reported in Euodiae Fructus. All the structures are shown in Fig. 10.

## Pharmacology

As a well-known medicinal plant in TCM, Euodiae Fructus has been validated to possess a diverse set of pharmacological properties, such as anticancer activity [96, 97], antibacterial activity [98], anti-inflammatory activity [45, 99, 100], insecticide activity [31], antinociceptive activity [101], and anti-diarrheal effect [102]. Evodiamine [1], rutaecarpine [4], and limonin [5], which are major compounds of this plant and display a variety of biochemical and pharmacological properties in the cancer, cardiovascular, central nervous system and so on, and it is possible to be developed as a promising lead compound for drug discovery. All the detailed information is shown in Table 3.

### Anticancer activity

In crude extracts, the methanol extract of Euodiae Fructus decreased the AP‐1 stimulator 12‐*O*‐tetradecanoylphorbol‐13‐acetate (TPA)‐induced AP‐1 transactivation in Chang/AP‐1 cells, with an EC_50_ value of 24.72 μg/mL [103]. Park et al. found that the 70% ethanol extract of Euodiae Fructus could induce apoptosis in HeLa cells with IC_50_ of about 45 μg/mL, which may be associated with a caspase-dependent cascade through activation of the intrinsic signaling pathway connected with AMP-activated protein kinase activation [96]. Another study showed that the 70% ethanol extract of Euodiae Fructus could inhibit the growth of benign prostatic hyperplasia-1 (BPH-1) epithelial cells by inhibiting proteins and antigens including 5*α*-reductase, proliferating cell nuclear antigen (PCNA), phosphor-ERK1/2, and cyclin D1 and by inhibiting cell viability dependently through the activation of caspase-3 and caspase-8 [97]. Additionally, ZJP aqueous extract exhibited its prominent therapeutic effects on hepatocellular carcinoma (HCC) mainly via the regulation of cell proliferation and survival though the EGFR/MAPK, PI3K/NF-κB, and CCND1 signaling pathways [104].

Numerous in vitro studies have reported that the isolated compounds of Euodiae Fructus display antitumor activities in several cancer cell lines, and the detailed information is presented in Table 3. Growing evidence demonstrates that evodiamine possesses anti-cancer activities both in vitro and in vivo by inhibiting proliferation, invasion and metastasis, inducing apoptosis of a variety of tumor cell lines, including colon cancer (HT-29, 26-L5, LoVo, COLO205 and HCT116), leukaemia (HL-60, CCRF-CEM, K562 and THP-1), hepatocellular carcinoma (Hep G2, Hepa1-6 and Hepa-1c1c7), lung cancer (H-460, A549), gastric cancer (N-87, AGS and SGC7901), renal carcinoma (Caki-1), breast cancer (MDA-MB-231), ovarian cancer cells (A2780/WT, A2780/PTXR, A2980, A2780CP, ES-2 and SKOV-3), prostate cancer (PC-3), melanoma (B16-F10, A375-S2), nasopharyngeal carcinoma (HONE1 and CNE1), glioblastoma (U87-MG, U87 and C6), urothelial cell carcinoma (5637 and HT1197), multiple myeloma (U266 and RPMI8226), cholangiocarcinoma (HuCCT-1 and TFK-1), cervical cancer (HeLa) cells etc. The related models are presented in Table 4.

**Table 4:** Pharmacological activities of Euodiae Fructus

Tested substance	Study	Cell Lines/Model	Active Concentration	References
Anti-Alzheimer’s disease				
Water extract	In vivo	Alzheimer’s disease (3xTg-AD) mice	400 mg/kg	[105]
Dehydroevodiamine	In vitro	SD rats Cerebellar Granule and Glial Cell	5 μM	[106]
Dehydroevodiamine	In vitro	Isolated rat brain	IC_50_ = 37.8 μM	[107]
	In vivo	Scopolamine-induced amnesia model	6.25 mg/kg	
Dehydroevodiamine	In vitro	Isolated rat brain with calyculin A-induced tau hyperphosphorylation	10, 100 and 200 µmol/L	[108]
Dehydroevodiamine	In vivo	Male Wistar rats with WT/GFX-induced tau hyperphosphorylation and memory impairment	6.25 and 12.5 mg/kg	[109]
Dehydroevodiamine	In vivo	Scopolamine-induced amnesia rat model; A*β*_1-42_ infused rat model	10 mg/ kg	[110]
Evodiamine	In vivo	C57BL/6 mice with ICV-STZ-induced experimental sporadic Alzheimer's disease	50 or 100 mg/kg	[111]
Evodiamine	In vitro	The SAMP8 and APP^swe^/PS^ΔE9^transgenic mouse in a C57BL/6 J	100 mg/kg	[112]
Evodiamine	In vitro In vivo	L-Glu-induced HT22 cell D-Gal and AlCl_3_-Induced AD Mice	5 to 40 µM 40 mg/kg	[113]
Neuroprotection activity				
Methanol extract	In vivo	Middle cerebral artery occlusion model	200 mg/kg	[114]
Evodiamine, Rutaecarpine, Dehydroevodiamine	In vitro	PC12 cell line with MPP + or H_2_O_2_-induced injury	20, 5 and 5 μM, respectively	[115]
Evodiamine	In vitro	Human prostate cancer cell line PC3, breast cancer cell line MCF7, and ovarian carcinoma cell A2780	10 μM	[15]
	In vivo	Adult male Sprague–Dawley rats model of paclitaxel-induced peripheral neuropathy	5 mg/kg	
Evodiamine, Rutaecarpine	In vitro	Human embryonic kidney 293 (HEK293) cells	10 and 50 μM	[116]
	In vivo	C57BL/6 mice of common peroneal nerve model or complete freund’s adjuvant model	0.3 and 0.29 mg/kg	
Rutaecarpine	In vivo	A middle cerebral artery occlusion rat model	5, 10 and 20 mg/kg	[117]
Anti-inflammatory and analgesic activity				
Ethanol extract	In vitro	A murine microglial cell line (BV2)	5–10 μg/mL	[118]
Rutaecarpine	In vitro	RAW 264.7 cells treated with lipopolysaccharide	IC_50_ = 31.62 ng/mL	[119]
Rutaecarpine	In vivo	Male C57BL/6 mice with sepsis	20 mg/kg	[120]
Rutaecarpine	In vitro	Bone marrow derived mast cells; COX-1 and COX-2 cDNA-transfected HEK293 cells	IC_50_ = 0.28 and 8.7 μM, respectively	[121]
	In vivo	Rat *λ*-carrageenan paw edema	10 mg/kg	
Rutaecarpine	In vivo	The DSS-induced acute colitis model	80 mg/kg	[122]
Evodiamine	In vitro	Human gastric mucosa cell line GES-1	0.5 μM and 1 μM	[123]
	In vivo	Ethanol-challenged experimental gastric ulcer model	20, 40 mg/kg	
Evodiamine	In vitro	RAW264.7 macrophage treated with zymosan	25 and 100 μM	[124]
	In vivo	A zymosan-induced generalized inflammation model	10 mg/kg	
Evodiamine	In vivo	Rat with adjuvant-induced arthritis	10, 20 and 40 mg/kg	[125]
Evodiamine	In vivo	Male SD rats with NTG-induced acute migraine	45 or 90 mg/kg	[126]
Evodiamine	In vivo	Male ICR mice with acetic acid-induced writhing	10–90 mg/kg	[127]
Limonin	In vitro	Normal colonic epithelial cells (NCM460)	2.5–160 μg/mL	[128]
	In vivo	The DSS-induced acute colitis model	40, 80 and 160 mg/kg	
Limonin	In vivo	Slc:ddy strain mice with AA-induced ear swelling	100 mg/kg	[69]
		Slc:ddy strain mice with carrageenin edema	30, 100 mg/kg	
Anti-cancer activity				
Ethanol extract	In vitro	HeLa human cervical carcinoma cells	IC_50_ = 45* μg*/mL	[96]
Ethanol extract	In vitro	The human BPH epithelial cell line BPH-1	6.25–200 μM	[97]
Methanol extract	In vitro	The human Chang liver cell line	EC_50_ = 24.72 μg/mL	[103]
Evodiamine	In vitro	Human breast cancer cell line MDA-MB-231	IC_50_ = 90 µM	[129]
	In vivo	Animal tumor xenograft model	10 mg/kg	
Evodiamine	In vitro	The human breast cancer cell lines MCF-7	1 × 10 ^−6^	[130]
Evodiamine	In vitro	The human SW1990 and PANC-1 PC cell lines	1–10 μM	[131]
	In vivo	Tumor-bearing nude mice	10, 20 and 30 mg/kg	
Limonin	In vitro	Breast cancer cell lines MCF-7 and MDA-MB-231	5 μM, 10 μM, 20 μM	[132]
Evodiamine	In vitro	Human colorectal carcinoma cells (COLO-205)	IC_50_ = 27.15 μM	[133]
Evodiamine	In vitro	Human colon cancer HCT116 cells	0.5–2 μM	[134]
	In vivo	Twenty athymic nude mice with colon cancer	10 mg/kg	
Evodiamine	In vitro	Sub-confluent LoVo cells	0.25–4 μM	[135]
	In vivo	Xenograft tumor model of human colon cancer	5, 10 and 20 mg/kg	
Evodiamine	In vitro	B16-F10, LLC and colon 26-L5 cell lines	IC_50_ = 2.4, 4.8 and 3.7 μM, respectively	[136]
Evodiamine	In vitro	The human colon cancer cell lines HT-29 cells and HCT-116 cells	IC_50_ = 6 μM	[137]
	In vivo	Female Balb/c nude mice were administered tail-vein injections of HCT-116 CRC cells	10 mg/kg	
Evodiamine	In vitro	A549 human lung cancer cells	IC_50_ = 1.3 μM	[138]
Evodiamine	In vitro	human NSCLC A549 and H1299 cell lines	IC_50_ = 41.13, 12.43 µM, respectively	[139]
Evodiamine	In vivo	Urethane-induced lung cancer mouse model	5 or 10 mg/kg	[139]
Evodiamine	In vitro	Two human NSCLC A549 and H1299 cell lines	1–16 μM	[140]
Limonin	In vitro	A549 human lung cancer cell line	50 and 75 µM	[141]
	In vivo	Swiss albino mice	50 mg/kg	
Evodiamine	In vitro	The hepatoma cell lines, HepG2 and Hepa1-6	0.1–10 μM	[142]
	In vivo	Hepa1-6 hepatoma-bearing animal model	10 and 20 mg/kg	
Evodiamine	In vitro	HepG2, SMMC-7721 and H22 cell lines	5 and 10 mmol/L	[143]
	In vivo	H22 xenograft mouse model	20 mg/kg	
Evodiamine	In vitro	Human HCC cell lines (HepG2 and SMMC-7721)	IC_50_ = 17.4 and 37.9 μM, respectively	[142]
	In vivo	BALB/c nude mice xenograft model	20 mg/kg	
Evodiamine	In vivo	Tumor xenograft models in nude mice	10 mg/kg	[144]
Evodiamine	In vitro	HCC cell lines (HepG2 and Bel-7402)	IC_50_ = 14.7 and 16 μM, respectively	[145]
	In vivo	Nude mice with xenograft tumors	10 mg/kg	
Limonin	In vitro	Human HCC cell lines HepG2, Huh7 and normal hepatic cell line L02	10, 20 and 40 μM	[146]
Evodiamine	In vitro	Gastric cancer cell lines AGS and SGC7901	IC_50_ = 5.06 and 3.54 μM, respectively	[147]
Evodiamine	In vitro	The BGC-823 human gastric carcinoma cell line	IC_20_ = 4 µmol/L	[148]
	In vivo	Male BALB/c mice gastric carcinoma xenograft model	10 mg/kg	
Evodiamine	In vitro	C6 and U87 glioma cells	IC_50_ = 4.3 and 3.7 μM, respectively	[149]
	In vivo	Athymic nude mice		
Evodiamine	In vitro	U87-MG malignant glioblastoma cell line	IC_50_ = 5.21 μM	[79]
Evodiamine	In vitro	Human bladder cancer cell lines 253 J and T24	IC_50_ = 1.90 and 2.14 μM, respectively	[150]
Evodiamine	In vitro	Human urothelial cell carcinoma cell lines, 5637 and HT1197	IC_50_ = 0.5 and 2.5 μM, respectively	[151]
Limonin	In vitro	Human ovarian cancer cell lines SKOV-3 and A2780	1–100 μM	[152]
Evodiamine	In vitro	Human ovarian cancer cell lines (SKOV-3, A2780, A2780CP, ES-2)	1–4 µM	[153]
Evodiamine	In vitro	Human ovarian epithelial cancer cell line HO-8910PM	IC_50_ = 3.94 μg/mL	[154]
Evodiamine	In vitro	Human renal carcinoma cell lines (786-O and Caki-1 cells)	IC_50_ = 23.707 μg/mL	[155]
Evodiamine	In vitro	A498 renal cell carcinoma cells	0.5–8 μM	[156]
	In vivo	Tumor xenograft implantation	30 mg/kg	
Evodiamine	In vitro	Human melanoma A375-S2 cells	15 μM	[157]
Evodiamine	In vitro	Murine fibrosarcoma L929	IC_50_ = 20.3 μM	[158]
Evodiamine	In vitro	The human osteosarcoma cell line 143B	0.5–2 μM	[159]
Evodiamine	In vivo	Xenograft tumor model of human osteosarcoma	20 and 50 mg/kg	[159]
Evodiamine	In vitro	Osteosarcoma U2OS cell and normal bone cells	IC_50_ = 6 µM	[160]
Evodiamine	In vitro	Human cholangiocarcinoma cell line HuCCT-1 and TFK-1	5–40 μM	[161]
	In vivo	Xenograft tumor bearing nude mice	20 mg/kg	
Evodiamine	In vitro	Human gastric cancer cell line SGC-7901	1 µM	[162]
Evodiamine	In vitro	Multiple myeloma U266 and RPMI8226 cells	400 μg/mL	[163]
	In vivo	Tumor Xenograft Model	400 mg/kg	
Evodiamine	In vitro	Human K562 myelogenous leukaemia cells, THP-1 acute monocytic leukaemia cells, CCRF-CEM leukaemic lymphoblast cells	IC_50_ = 34.43, 58.42 and 4.70, respectively	[164]
Dihydroevocarpine	In vitro	MV‐4–11, HS‐5, KASUMI‐1 and HL‐60 cell lines	IC_50_ = 5.7, 8.7, 8.1 and 4.9 μM, respectively	[165]
	In vivo	Acute myeloid leukemia acute xenograft model	10 mg/kg	
Anti-cardiovascular disease activity				
Aqueous extract	In vitro	Aorta strips of male Wistar rats	1 × 10^–6^–3 × 10^–4^ g/mL	[65]
Aqueous extracts	In vivo	Male Hartley guinea pigs	1,000 μg/mL	[166]
Rutaecarpine	In vivo	ADP-induced acute pulmonary thrombosis in mice	25 and 50 μg/kg	[167]
Rutaecarpine	In vitro	Human platelet-rich plasma	40–200 μM	[168]
Rutaecarpine	In vitro	Human platelet suspensions	60 and 100 μM	[169]
Rutaecarpine	In vivo	Spontaneously hypertensive rats	10, 20 or 40 mg/kg	[170]
Rutaecarpine	In vitro	HUVECs with high glucose-induced GJ dysfunction	0.1, 0.3 and1 µM	[171]
Rutaecarpine	In vitro	Hypoxia-induced human pulmonary artery smooth muscle cells	IC_50_ = 43.5 µmol·L^−1^	[172]
Rutaecarpine	In vitro	Ang II-induced VSMC proliferation	0.3–3.0 μM	[173]
Rutaecarpine	In vitro	Ox-LDL-induced VSMCs dysfunction	10 µM	[174]
Rutaecarpine	In vitro	Cultured THP-1 exposed to ox-LDL	0.1, 0.3 and 1 μM	[175]
Rutaecarpine	In vitro	Ox-LDL-induced HUVEC-12 dysfunction	0.1, 0.3 and 1 μM	[176]
Rutaecarpine	In vivo	C57/BL6 ApoE ˉ/ˉmice with atherosclerosis	10, 20, and 40 mg/kg	[177]
Evodiamine	In vitro	Platelet-derived growth factor-BB induced-rat VSMCs migration	0.1 and 0.5 µM	[178]
Evodiamine	In vitro	Human umbilical vein endothelial cells with high glucose -induced proliferation	4 μM	[179]
Evodiamine	In vitro	HUVECs with high free fatty acids; THP-1 cells	2.5 μM	[180]
Rutaecarpine	In vitro	The thoracic aorta and the superior branch of mesenteric artery of Rats	10^–7^–10^–5^ M	[181]
	In vivo	Male Wistar rats	30, 100 or 300 μg/kg	
Rutaecarpine	In vitro	Isolated primary ventricular cardiomyocytes Ang II‐induced cardiac hypertrophy	10 μM	[182]
Rutaecarpine	In vivo	AAC‐induced cardiac hypertrophy model	20 and 40 mg/kg	[182]
Rutaecarpine	In vivo	Balloon-injured rat artery model	50 and 75 mg/kg	[183]
Rutaecarpine	In vitro	Isolated perfused heart of Guinea pigs	0.3 or 1 μM	[184]
Rutaecarpine	In vivo	Male Wistar rats with myocardial ischemia–reperfusion injury	100 or 300 μg/kg	[185]
Rutaecarpine	In vivo	Male Wistar rats with myocardial ischemia–reperfusion injury	0.1 ml/kg	[186]
Evodiamine	In vitro	The isolated guinea pig heart model	0.3 or 1 µM	[187]
Evodiamine	In vivo	Male SD rats with myocardial I/R injury	30 or 60 μg/kg	[188]
Evodiamine	In vitro	TGF-*β*1-induced neonatal rat cardiac fibroblasts	1, 5, and 10 μM	[189]
Evodiamine	In vitro	Angiotensin II-induced rat cardiomyocyte hypertrophy	0.3, 3 μM	[190]
Evodiamine	In vivo	Male C57BL/6 mice with isoproterenol-induced cardiac fibrosis	50 and 100 mg/kg	[191]
Anti-obesity and anti-diabetic activity				
Evodiamine	In vitro	3T3-L1 preadipocytes; 3T3-L1 adipocytes	100 μM	[192]
Evodiamine	In vivo	Male Sprague–Dawley rats	40 mg/kg	[193]
Evodiamine	In vitro	3T3-L1 cells	20 μM	[194]
	In vivo	Obese/diabetic KK-Ay mice	3 mg/kg	
Rutaecarpine, Evodiamine	In vitro	Human hepatoma HepG2 cells	25 and 10 μM, respectively	[195]
	In vivo	C57BL/6 J, db/db, ob/ob and CAR^−/−^mice	10 mg/kg	
Rutaecarpine	In vitro	Cultured skeletal muscle cells	20–180 μM	[196]
	In vivo	The fat-fed/STZ rat model	25 mg/kg	
Antibacterial activity				
Ethanol extract	In vitro	Bacterial strains (*Staphylococcus aureus* ATCC 25,923, ATCC 6538, *Streptococcus pyogenes* Δ-68, *Escherichia coli* ATCC 11,229, *Proteus mirabilis* ATCC 14,159, *Pseudomonas aeruginosa* ATCC 27,853) and the yeast *Candida albicans* CBS 5982	MIC = 1.0, 0.5—1.0, 0.25, 1.0, 1.0, 1.0 and 0.5 mg/mL, respectively	[98]
The essential oils	In vitro	*Bacillus subtilis* and *Staphylococcus aureus*	MIC = 3.2–6.4 mg/mL	[91]
Rhetsinine	In vitro	*Xanthomonas oryzae* pv. *oryzae*, *Xanthomonas oryzae* pv. *oryzicola* and *Xanthomonas campestris* pv. *campestris* strains	EC_50_ = 3.13, 14.32 and 32.72 nmol, respectively	[29]
Evodiamine	In vitro	Mouse macrophage cell line J774A.1; The lipopolysaccharide-primed macrophages	1.25–5.0 μM	[197]
	In vivo	Female C57BL/6 mice with bacterial infection	10 or 20 mg/kg	
Insecticidal activity				
The essential oil	In vitro	Maize weevils, *Sitophilus zeamais* and red flour beetles *Tribolium castaneum*	LC_50_ = 36.89, 24.57 and 57.31 mg/L air, respectively	[93]
Ethanol extract, Evodiamine	In vitro	*Meloidogyne incognita*	LC_50_ = 131.54 μM and LC_50_ = 73.55 μM	[30]
Ethyl acetate extract	In vivo	Goldfish-*Gyrodactylus kobayashii* Model	EC_50_ = 24.0 mg/L	[198]
Petroleum ether extract	In vivo	Goldfish-*Gyrodactylus kobayashii* Model	EC_50_ = 71.9 mg/L	[198]
Methanol extract	In vivo	Goldfish-*Gyrodactylus kobayashii* Model	EC_50_ = 40.9 mg/L	[198]
Evodiamine, rutaecarpine	In vitro	*Drosophila melanogaster* Meigen	LC_50_ = 0.30 and 0.28 μM, respectively	[199]
Bone metabolism regulation				
Evodiamine	In vitro	Isolatied mice bone marrow macrophage	5 and 15 μg/mL	[200]
Evodiamine	In vitro	Isolated C57BL/6 mice bone marrow macrophage‐derived osteoclast	1–10 μM	[201]
	In vivo	Ovariectomized (OVX) mouse model	10 mg/kg	
Evodiamine	In vivo	Zebrafish with dexamethasone-induced osteoporosis	50, 100 mg/kg	[202]
Rutaecarpine	In vitro	Bone marrow-derived macrophages	0.1–10 μM	[203]
Limonin	In vitro	Osteoblastic MC3T3-E1 cells	5–40 μM	[204]
	In vivo	Ovariectomised (OVX) animal model	250 mg/kg	
Hepatorenal protection				
Evodiamine	In vitro	Hepatic stellate cells	2–20 μM	[205]
	In vivo	Carbon tetrachloride (CCl_4_)-induced liver fibrosis in rats	15 and 25 mg/kg	
Evodiamine	In vivo	Male Wistar Albino rats with Renal ischemia/reperfusion (I/R) injury	10 mg/kg	[206]
Evodiamine	In vitro	The NRK-52E rat proximal tubular cell line	10 and 20 mg/kg	[207]
	In vivo	Male SD rats with lipopolysaccharide-LPS-induced acute kidney injury		
Rutaecarpine	In vitro	HepG2 cells with t-BHP-induced hepatotoxicity	1–10 μM	[208]
	In vivo	Male ICR mice with t-BHP-induced hepatotoxicity	5 mg/kg	
Rutaecarpine	In vivo	The IRI rat model	30, 60 mg/kg	[209]
Limonin	In vitro	L-02 cells	10, 25, 50 μM	[210]
	In vivo	Acetaminophen-induced liver injury model	40, 80 mg/kg	
Other activity				
50% Ethanol extract	In vivo	Castor oil-induced diarrhea	ID_50_ = 54 mg/kg	[102]
Evodiamine	In vivo	A rat model of chronic unpredictable mild stress	20 mg/kg	[211]
Evodiamine	In vitro	HEK 293 cells with capsaicin-induced currents	10 μM	[212]
	In vivo	Male adult SD rats with capsaicin-induced thermal hyperalgesia	100 μM, 50 *μ*L	
Evodiamine	In vivo	KCN-induced anoxia model in mice	50 mg/kg	[213]
Evodiamine	In vivo	The male ICR mice and male SD rats	50 mg/kg	[214]
Evodiamine	In vitro	The virus stocks of IAV subtypes	1.54–12.5 μg/mL	[215]

### Antibacterial and antifungal activity

Euodiae Fructus has been used to treat infection-related diseases including diarrhea, beriberi and oral ulcer for a long time due to its antibacterial and antifungal activities. The ethanol extract of Euodiae Fructus inhibited the growth of *Staphylococcus aureus* ATCC 25923, *Staphylococcus aureus* ATCC 6538, *Streptococcus pyogenes* Δ-68, *Escherichia coli* ATCC 11229, *Proteus mirabilis* ATCC 14159, *P. aeruginosa* ATCC 27853, and *Candida albicans* CBS 5982, with minimum inhibitory concentration (MIC) values of 1.0, 0.5–1.0, 0.25, 1.0, 1.0, 1.0, and 0.5 mg/mL, respectively after 24 h of incubation in Muller-Hinton broth [98]. Another study showed that the 95% methanol extract showed inhibitory activity against *Helicobacter pylori* ATCC 49503 with MIC value of 25 μg/mL, and inhibited the urease activity in H. pylori via inhibiting the ureB expression [216]. Moreover, Liu et al. found that essential oils of Euodiae Fructus show the most potent activities against *Bacillus subtilis* and *Staphylococcus aureus*, with the largest inhibition zone diameters of 17.9 and 12.2 mm, respectively, and the MIC values of 3.2–6.4 mg/mL [91].

In isolated compounds, the two novel alkyl methyl quinolone alkaloids (compounds **80–81**) (AM quinolones) shown highly selective antimicrobial activity against *H. pylori* without harmful adverse effects against other intestinal flora [56], thereby being a candidate for use in eradication therapy for *H. pylori *in vitro and vivo [217]. In addition, evodiamine was able to augment the NLRP3 inflammasome activation by inducing acetylation at K40 residue of α-tubulin, thus conferring intensified innate immunity against bacterial infection [197].

### Anti-inflammatory and analgesic activity

Euodiae Fructus has been used in TCM for the treatment of inflammation-related disorders such as gastrointestinal disorders (gastric ulceration, ulcerative colitis and dysentery), headache, postpartum hemorrhage, amenorrhea and dermatitis [121]. Numerous studies have demonstrated that dysregulation of nuclear factor-kappa B (NF-кB) pathways and inflammatory factors, such as TNF-*α*, IL-1*β*, IL-6 and NO, etc. play important roles in inflammatory responses [218].

#### Anti-inflammatory activity

The water extract of Euodiae Fructus could enhance the gastric mucosal barrier and promote the synthesis of NO in gastric mucosa, which has a significant protective effect toward ethanol-induced gastric injury in rats [219]. Ko et al. showed that the ethanol extract of Euodiae Fructus display potent antioxidative effects against both phorbol-12-myristate-13-acetate (PMA)- and *N*-formyl-methionyl-leucyl-phenylalanine (fMLP)-induced ROS production in neutrophils with respective IC_50_ values of 2.7 and 3.3 μg/mL and also inhibit lipopolysaccharide (LPS)-induced NO production with an IC_50_ of around 0.8 μg/mL, suggesting that the ethanol extract exhibited anti-inflammatory activities which could be partially explained by inhibiting NADPH oxidase-dependent ROS and/or iNOS-dependent NO production in activated inflammatory cells [118]. In another study, Euodiae Fructus and its active components may be useful in influenza virus infection-related inflammatory disorders by suppressing novel influenza A (H1N1)-induced chemokines (RANTES and MCP-1) production and blocking chemokine-attracted leukocytes recruitment [100].

In isolated compounds, results have showed that the anti-inflammatory effect of rutaecarpine is partly ascribed to the diminution of prostaglandin (PG) production through inhibition of arachidonic acid release in the RAW 264.7 [119]. In other studies, rutaecarpine ameliorated sepsis-induced peritoneal resident macrophages apoptosis and inflammation responses through inhibition of endoplasmic reticulum stress-mediated caspase-12 and NF-κB pathways [120], improved imiquimod-induced psoriasis-like dermatitis through effects on pDC- and Th17-associated cytokines via modulation of NF-κB and toll-like receptor 7 (TLR7) signaling [220], and ameliorated dextran sulfate sodium (DSS)-induced ulcerative colitis (UC) via inhibiting KEAP1-NRF2 interaction to activate NRF2 [122]. Similarly, limonin was reported to improve the prognosis of DSS-induced UC mainly through downregulating p-STAT3/miR-214 levels [128]. Moreover, evodiamine could improve antioxidant and anti-inflammatory status through Rho/NF-κB pathway, which possibly exerted a gastro-protective effect against gastric ulceration [123]. In vitro and vivo, evodiamine was able to protect against zymosan-induced inflammation and DSS-induced murine experimental colitis by inactivating the expression of pro-inflammatory cytokines (IL-1*β*, IL-6 and TNF-*α*), NF-κB signal pathway [124] and NLRP3 inflammasome [221], and the antiarthritic effect of evodiamine might be associated with its repression of synovial inflammation and regulation of Treg and Th17 differentiation [125].

#### Antinociceptive activity

Moreover, it has been reported that oral administration of 50 or 200 mg/kg 70% methanol extract of Euodiae Fructus has an antinociceptive effect on acetic acid induced-writhing responses, and the mode of action may be mediated by its anti-inflammatory action [101]. In vivo, limonin (30 or 100 mg/kg) possessed an antinociceptive effect and the effect may be accompanied by an anti-inflammatory action [69]. In other studies, evodiamine could reduce capsaicin-induced currents significantly in vitro and suppress capsaicin-induced thermal hyperalgesia in rats, which may be due to the activation and subsequent desensitization of TRPV1 in sensory neurons [212], and it could also inhibit the migraine-like pain response possibly due to the regulation of nNOS and suppression of the AMPA receptor GluA1 [126].

### Anti-cardiovascular disease activity

Several studies have demonstrated that Euodiae Fructus has anti-cardiovascular activities, such as vasoconstrictive and vasodilator effects, anti-atherosclerosis, anti-platelet aggregation, anti-thrombus, anti-arrhythmia and cardioprotective effects [3].

#### Vasoconstrictive and vasodilator activity

It has been found that Euodiae Fructus (1 × 10^−6^–3 × 10^–4^ g/mL) has constrictive effects on rat aorta via adrenergic *α*_1_ receptors and serotonergic (5-HT_1D_ and 5-HT_2A_) receptors [65], and the effect toward calcium channel on the membrane also played important roles [222]. In other investigations, rutaecarpine produced a fully (100%) NO-dependent vasodilatation in rat aorta, whereas dehydroevodiamine and evodiamine produced a partially endothelium-dependent effect, 10% and 50%, respectively. Furthermore, multiple-action mechanisms, including endothelium dependence, α_1_-adrenoceptor blockade, K^+^ channel activation, and Ca^2+^ channel blockade were probably involved in the vasorelaxant effects of dehydroevodiamine [223]. In vivo and vitro, the depressor and vasodilator effects of rutaecarpine were related to stimulation of endogenous CGRP release via activation of vanilloid receptors [181, 224].

#### Modulatory effects on VSMCs function and intimal hyperplasia

Results showed that evodiamine suppressed oxidative stress and inflammatory responses due to high free fatty acids and high glucose in human umbilical vein endothelial cells (HUVECs) via inhibiting the upregulated expression of P2X4R signaling pathway [179] and P2X_7_ receptor [180], respectively. Further investigations have shown that a promising anti-atherogenic effect of evodiamine through attenuation of vascular smooth muscle cells (VSMCs) migration by suppressing cell cycle progression, p38 MAPK and Erk1/2 activation, and ROS generation [178], and the activation of PPARγ also plays important role [225]. It was worth noting that rutaecarpine could modulate Cx (theroprotective Cx37 and atherogenic Cx43) expression through TRPV1/[Ca^2+^]i/CaM/NF-κB signal pathway [174] in monocytes to enhance its antiadhesive properties [171, 175], thereby preventing VSMCs dysfunction induced by ox-LDL [176]. Additionally, rutaecarpine inhibited Angiotensin II-induced proliferation in VSMCs partly through the modulation of NO signaling pathways and other related molecules (HRG-1 and c-myc) [173]. Moreover, rutaecarpine (10, 20, and 40 mg/kg) suppressed atherosclerosis in ApoE^−/−^ mice through upregulating ABCA1 and SR-BI within reverse cholesterol transport (RCT) [177], and it could also promote NO production and inhibit ERK2 signal transduction pathways to inhibit the balloon injury-induced carotid intimal hyperplasia in rats [183].

#### Anti-platelet activity

“Goshuyuto” at the concentration of 1000 μg/mL inhibited collagen-induced platelet hyper-aggregation to the same degree as aspirin at the concentration of 100 μM [166]. Rutaecarpine was also able to display an anti-platelet effect in vivo [167], and the mechanism was investigated by inhibition of thromboxane formation and phosphoinositide breakdown [168]. Further investigation has shown that rutaecarpine inhibits agonists-induced platelet aggregation in human platelets, probably by inhibition of phospholipase C activity, leading to reduce phosphoinositide breakdown, followed by inhibition of thromboxane A_2_ formation and [Ca^2+^]_i_ mobilization [169]. In another study, rutaecarpine has been seen to exert both antihypertensive and anti-platelet effects by stimulating the synthesis and release of CGRP in spontaneously hypertensive rats (SHR), and CGRP-mediated antiplatelet effect was related to inhibit the release of platelet-derived tissue factor [170].

#### Anti-arrhythmia activity

It has also been found that evodiamine and rutaecarpine induce the positive inotropic and chronotropic effects on the guinea-pig isolated right atria through their interaction with vanilloid receptors and the resultant release of CGRP [226, 227]. Additionally, dehydroevodiamine (0.1–0.3 μM) could depress trigger arrhythmias in Ca-overloaded guinea-pig cardiac myocytes through inhibiting *I*_Na_, *I*_ti_ and, to a smaller extent, *I*_Ca_, while increasing the intracellular pH (pH_i_) and Na^+^–H^+^ exchanger (NHE) activity [228].

#### Regulatory effects on cardiac injury

Yi et al. found that the protective effects of rutaecarpine on cardiac anaphylactic injury or ischemia–reperfusion injury were related to inhibition of TNF-*α* production by stimulation of CGRP release [184], and the involvement of capsaicin-sensitive sensory nerves also played important roles [185], and the inhibition of Nox4‐ROS‐ADAM17 pathway and over‐activation of ERK1/2 might be associated with the beneficial role of rutaecarpine in hypertensive cardiac hypertrophy [182]. Moreover, evodiamine (0.3 and 3 μM) significantly attenuated Ang II-induced cardiomyocyte hypertrophy in vitro, and this effect is partly due to the promotion of NO production, the reduction of [Ca^2+^]_i_ concentration, and the inhibition of CaN and ERK-2 signal transduction pathways [190], and it could also prevent cardiac fibroblasts from activation into myofibroblast and protect HUVEC against endothelial to mesenchymal transition (EndMT) probably by inhibition of canonical [189] and non-canonical TGF*β* signaling [191].

### Neuroprotective activity

A wide spectrum of pharmacological experiments indicated that Euodiae Fructus and its isolated compounds exerted a neuroprotective effect against ischemic injury, neuropathic pain, nerve inflammation, neurodegenerative disorders such as Alzheimer’s disease (AD), etc. The methanol extract of Euodiae Fructus (200 mg/kg) was able to have a protective effect against ischemia-induced neuronal and cognitive impairment [114]. In a MDCK-pHaMDR cell monolayer model, evodiamine and rutaecarpine entered the blood–brain barrier (BBB) by passive diffusion and promoted the absorption of dehydroevodiamine probably by inhibiting P-gp, while dehydroevodiamine showed moderate permeability through BBB by P-gp mediated efflux. Moreover, the above three alkaloids have been confirmed to exhibit neuroprotective effects on MPP^+^ or H_2_O_2_-injured PC12 cells [115]. In other studies, evodiamine (10 μM) and rutaecarpine (50 μM) reduced peripheral hypersensitivity and anxiety in mice with nerve injury or inflammation via TRPV1 [116]. Moreover, evodiamine could ameliorate paclitaxel-induced neuropathic pain by inhibiting inflammatory response and activating mitochondrial anti-oxidant functions [15], and induced JNK-mediated protective autophagy in astrocytes through TRPV1-dependent signaling and an influx of extracellular calcium, which may provide a possible option for ischemic stroke treatment [229]. Additionally, rutaecarpine improved neuronal injury, inhibited apoptosis, inflammation and oxidative stress in rats with cerebral ischemia–reperfusion (CI/R) by regulating the expression of ERK1/2 and Nrf2/HO-1 pathway [117].

Besides the above functions on the nervous system, Euodiae Fructus and its isolated compounds could also potentially be developed as an alternative therapeutic agent for the management of AD. Cai et al. demonstrated that the water extract of Euodiae Fructus significantly ameliorated learning and memory deficits in Morris water maze tests, and in 3xTg AD mice, it could also decrease A*β* deposits and increase NeuN-positive cells by upregulating the expressions of Brain neurotrophic derived factor (BDNF) and tyrosine kinase B (TrkB) [105]. Evodiamine (100 mg/kg) significantly alleviated the impairments of learning ability and memory in transgenic mouse models [112], and inhibited glial cell activation and neuroinflammation (IL-1*β*, IL-6, TNF-*α*, and COX-2 levels) in the hippocampus by increasing the activity of AKT/GSK-3β signaling pathway and inhibiting the activity of NF-κB [111]. Further study has revealed that evodiamine exerts a protective effect against AD by modulating oxidative stress and reducing the apoptosis rate in vitro and vivo [113]. Additionally, dehydroevodiamine could inhibit acetylcholinesterase activity with IC_50_ value of 37.8 μM and show antiamnesic effect due to the combined effects of acetylcholinesterase inhibition and the known cerebral blood flow enhancement [107], and it could also suppress WT/GFX-induced overactivation of GSK-3 to improve spatial memory impairment and tau hyperphosphorylation in vivo [109], and its underlying mechanism might involve a decreased inhibitory phosphorylation of PP-2A at Tyr307 [108], and the protective effects on cognitive impairment might be related to its antioxidant activity, inhibition of neurotoxicity and intracellular calcium in memory-impaired rat models [110].

### Anti-obesity and anti-diabetic activity

#### Anti-obesity activity

It has been reported that ruteacarpine and evodiamine [193] reduce food intake and bodyweight gain by improving orexigenic sensitivity through the inhibition of neuropeptide Y (NPY) and agouti-related protein (AgRP) mRNA expression and peptide expression [230]. Moreover, evodiamine, as a vanilloid receptor agonist, could simultaneously induce heat loss and heat production and dissipate food energy, preventing the accumulation of perivisceral fat and the body weight increase [231], and activate AMP-activated protein kinase (AMPK) and adiponectin multimerization in 3T3-L1 adipocytes, which was associated with the activation of Ca^2+^-dependent PI3K/Akt/ CaMKII-signaling pathway [192].

#### Anti-diabetic activity

Furthermore, rutaecarpine and evodiamine were able to suppress gluconeogenesis and lipogenesis through their activation of the constitutive androstane receptor (CAR) in vitro and vivo, thus having a therapeutic potential for the treatment of hyperglycemia and type 2 diabetes [195]. Evodiamine improved glucose tolerance and reduced insulin resistance in obese/diabetic mice, which was possibly related to inhibition of mammalian target of rapamycin (mTOR)- S6 protein kinase (S6K) signaling and insulin receptor substrate 1 (IRS1) serine phosphorylation in adipocytes [194]. An additional study demonstrated that rutaecarpine could regulate IRS-1/PI3K/Akt signaling pathway in liver and AMPK/ acetyl-CoA carboxylase2 (ACC2) signaling pathway in skeletal muscles to ameliorate hyperlipidemia and hyperglycemia in fat-fed, streptozotocin-treated rats [196].

### Insecticidal activity

In recent years, plant-based, environmentally friendly and biodegradable natural insecticides have received renewed attention as vector control agents, and some research have demonstrated that Euodiae Fructus exhibit insecticidal activity [232]. Lian et al. screened different extracts of Euodiae Fructus with anthelmintic activity against *Gyrodactylus kobayashii* (Monogenea) in goldfish, indicating that the ethyl acetate, the petroleum ether and methanol extracts had better anthelmintic efficacy, with EC_50_ values of 24.0, 71.9 and 40.9 mg/L, respectively, after a 48-h exposure, whereas the water extract of Euodiae Fructus had the weakest anthelmintic efficacy of 25.6% at 800.0 mg/L [198]. Moreover, the essential oil of Euodiae Fructus was found to possess insecticidal activity against maize weevils, *Sitophilus zeamais* and red flour beetles *Tribolium castaneum* with LC_50_ values of 36.89, 24.57 and 57.31 mg/L air, respectively [93]. Further study has shown that evodiamine and rutaecarpine showed insecticidal activity against larvae of D melanogaster with LC_50_ values of 0.30 and 0.28 μmol/mL diet respectively [199]. In another investigations, evodiamine, rutaecarpine, and wuchuyuamide I have been reported to exhibit strong larvicidal activity against the early fourth instar larvae of *A. albopictus* with LC_50_ values of 12.51, 17.02, and 26.16 μg/mL, respectively, and the ethanol extract, limonin and evodol also possessed larvicidal activity against the Asian tiger mosquitoes with LC_50_ values of 43.21, 32.43 and 52.22 μg/mL, respectively [31]. Liu et al. showed that evodiamine (LC_50_ = 73.55 μg/mL) and rutaecarpine (LC_50_ = 120.85 μg/mL) exhibit stronger nematocidal activity against *M. incognita* than the crude ethanol extract of Euodiae Fructus (LC_50_ = 131.54 μg/mL) [30]. Additionally, rhetsinine was found to show potential as a pesticide and exhibited excellent inhibition against *Xanthomonas oryzae* pv. *oryzae*, *Xanthomonas oryzae* pv. *oryzicola*, and *Xanthomonas campestris* pv. *campestris*, with respective EC_50_ values of 3.13, 14.32, and 32.72 nmol in vitro [29]. Taken together, these results indicated that the ethanol extract of Euodiae Fructus and several isolated constituents have a good potential as a source for insecticidal activity, and further research is needed to determine its safety to human body and environment.

### Hepatorenal protection

Consistent with traditional applications, Euodiae Fructus was reported to affect the liver and kidney [233]. Jin et al. reported that rutaecarpine augmented cellular antioxidant defense capacities through CaMKII-PI3K/Akt-dependent HO-1 induction via the Nrf2/ARE signaling pathway, thereby protecting cells from oxidative damage in hepatocytes [208]. It has been found that evodiamine (15 and 25 mg/kg) has an antifibrosis effect in CCl_4_-induced liver fibrosis and reduces hepatic stellate cells (HSCs) proliferation and collagen metabolism in vitro through downregulation of relative expression of TGF*-β*1, p-Smad 2/3, and *α*-SMA [205]. In other investigation, limonin alleviated acetaminophen-induced hepatotoxicity by activating Nrf2 antioxidative signals and inhibiting NF-κB inflammatory response via upregulating Sirt1 [210]. For the kidney, recent researches showed that a number of protective roles against I/R damage [206], LPS-induced acute kidney injury and cytotoxicity [207] due to the antioxidative, anti‐inflammatory and antiapoptotic properties of evodiamine. Additionally, Wang et al. showed that rutaecarpine be an effective compound for the prevention and treatment of renal ischemia–reperfusion injury (IRI), and its mechanism might be related to inhibition of JNK/p38MAPK signaling pathway and interference of oxidative stress response [209].

### Anti-osteoporosis activity

Rutaecarpine significantly inhibited osteoclastogenesis and prevented bone resorption of bone marrow-derived macrophage (BMM)-derived osteoclasts through decreasing the protein level of nuclear factor of activated T cells cytoplasmic-1 (NFATc1) and the phosphorylation of other signaling pathways during the osteoclast differentiation [203]. Moreover, evodiamine was reported to inhibit the formation of osteoclasts via blocking the RANKL-induced activation of ERK and c-Fos as well as the induction of NFATc1[200], and the underlying mechanism might also be related to inhibit the activation of the NF‐κB and calcium signalling pathways [201], and in Zebrafish, evodiamine was found to prevent osteoporosis by reversing the imbalance of bone formation/bone resorption and activating MMP3-OPN-MAPK pathway signal [202]. Additionally, limonin stimulated alkaline phosphatase (ALP) activity and enhanced the expression of osteoblast differentiation gene markers by regulating ERK and P38 signals in osteoblastic MC3T3-E1 cells, and inhibited the reduction of bone mass and promote the increase of bone mineral density in ovariectomized rats [204].

### Other activity

Apart from the summarized pharmacological activities mentioned above, the isolated constituents or crude extracts of Euodiae Fructus also involve other bioactivities including anti-diarrheal effect, antiallergic effect, antianoxic activity, antidepressant-like activity, antiviral activity, anti-ovotoxicity effect, etc. It has been reported that Euodiae Fructus has both anti-transit and anti-diarrheal effects with comparable ID_50_ (the dose for 50% inhibition) values of 54 ± 7 and 76 ± 17 mg/kg and the anti-diarrheal effect of Euodiae Fructus may be associated with its anti-transit [102]. In vitro and vivo, Euodiae Fructus and its constituents (evodiamine and rutaecarpine) might inhibit the biosynthesis of anaphylaxis-related cytokines (TNF-*α* and IL-4) in mast cells and basophils, suggesting that they might be effective for IgE-induced allergic diseases such as atopic dermatitis and rhinitis [214]. Other studies have demonstrated that the involvement of cholinergic mechanism plays important roles in the antianoxic potential of evodiamine in the KCN-induced anoxia model [213, 234]. Moreover, the antidepressant-like effect of evodiamine on chronic unpredictable mild stress rats probably by modulating effects on the monoamine transmitters and brain-derived neurotrophic factor (BDNF)-tropomyosin-related kinase B receptor (TrkB) signaling in the hippocampus [211]. Dai et al. showed that evodiamine could significantly inhibit the replication of anti-influenza A virus (IAV), the accumulation of LC3-II, p62 and EGFP-LC3, the formation of the Atg5-Atg12/Atg16 heterotrimer, the expressions of Atg5, Atg7 and Atg12, and the cytokine release of TNF-*α*, IL-1*β*, IL-6 and IL-8 after IAV infection, meanwhile, the inhibition of IAV-induced autophagy by evodiamine was also dependent on its action on the AMPK/TSC2/mTOR signal pathway [215]. In addition, the water extract of Euodiae Fructus could activate Akt to protect ovary cells against 4-vinylcyclohexene diepoxide-induced ovotoxicity, which indicates that Euodiae Fructus may help prevent premature ovarian failure or unexplained infertility caused by environmental factors [13]. Interestingly, a recent study has shown that aqueous extract of Euodiae Fructus and evodiamine could improve caffeine-induced sleep and excitation behaviors, at least in part, through the *γ*-aminobutyric acid (GABA)_A_-ergic system, these results suggest a potential therapeutic agent to treat insomnia or sleep problems related to caffeine intake [235].

## Toxicity

According to China’s most ancient herbal medicine book “Shen Nong’s Herbal Classic” and 2020 Edition of Chinese Pharmacopoeia, the mild toxicity of Euodiae Fructus has been noted. In recent years, it has been reported that the cases of patients with chronic esophagitis, excessive use of Euodiae Fructus could cause stomach pain, vomiting, blurred vision and other toxic symptoms [236, 237], and cause liver toxicity to the human body [238, 239]. Modern researches in vitro and in vivo have shown that the crude extract and several compounds isolated from Euodiae Fructus have been reported to exert hepatic injury, CYP inhibition, and to induce proarrhythmic cardiotoxicity when used in high doses as described in Table 5, and the details will be further discussed below.

**Table 5:** Toxicity of Euodiae Fructus and its constituents

Parameter	Study	Tested substance	Cell lines/model	Dosage of administration	Activity/Mechanism(s) of Action	Reference
Hepatotoxicity	In vivo	Aqueous extract	Adult male rats of SD strain	6, 12, 24 g/kg	Resulted in ATP depletion and CytC release, finally trigger cell death signaling	[241]
Hepatotoxicity	In vitro	Rutaecarpine	Isolated rat hepatocytes	10, 30, 100 and 300 mM	Inhibited the activities of CYPs and CYP1A2	[242]
Hepatotoxicity	In vitro	Rutaevin	Mice serum	–	Increased the activities of ALT and AST	[244]
Cardiovascular toxicity	In vivo	Evodiamine	Zebrafish	LC_10_ = 354 ng/mL	Increased lactate dehydrogenase release and maleic dialdehyde levels, and reduced superoxide dismutase activity	[250]
	In vitro		Neonatal rat cardiomyocytes	IC_50_ = 28.44 µg/mL		
Proarrhythmic effects	In vitro	Dehydro-evodiamine	HEK 293 cells	IC_50_ = 253.2 nM	Inhibited hERG channels	[249]
	In vitro		cAVB dog cardiomyocytes	0.01–10 μM	APD prolongation, increase in STV and the incidence of EADs	
	In vivo		Anesthetized rabbits	0.5 mg/kg	Induced TdP arrhythmias in 2 out of 8 animals	

In acute toxicity test, histopathological analysis revealed that Euodiae Fructus caused morphological changes in the liver, but no other main organs [240]. Cai et al. reported that oral gavaging of water decoction at dose of 6, 12 and 24 g/kg for 15 days in rats could increase malondialdehyde (MDA) level, and decrease the MnSOD activity and glutathione (GSH) levels reduction, followed by causing oxidative damage, finally resulting in adenosine triphosphate (ATP) depletion and cytochrome C (CytC) release, triggering cell death signaling pathways, which are all partial hepatotoxicity mechanisms of Euodiae Fructus [241], In another study, rutaecarpine might be a mechanism-based inhibitor of CYP1A2, and its potential hepatotoxicity might be related to reactive metabolites, and GSH trapping might be a detoxication route [242]. Furthermore, in vitro, rutaecarpine, evodiamine, and dehydroevodiamine significantly activated aryl hydrocarbon receptor (AHR), with an efficacy order of rutaecarpine > dehydroevodiamine > evodiamine, and ligand-docking analysis predicted that the methyl substitute at the N-14 atom was a key factor affecting AHR activation. The above three indole alkaloids were not hepatotoxic in vivo at the doses used. However, rutaecarpine and dehydroevodiamine disrupted bile acid homeostasis in an AHR-dependent manner, evodiamine failed to activate AHR due to its poor absorption in mice [243]. A recent study has revealed that rutaevin was shown to increase the activities of alanine aminotransferase (ALT) and aspartate aminotransferase (AST) in mice serum, suggesting the potential hepatotoxicity of rutaevin, and the potential mechanism was that rutaevin was converted into a electrophilic BDA intermediate by CYP3A4 [244]. Moreover, it has been reported that dihydrorutaecarpine (**5**), 6-acetoxy-5-epilimonin (**146**), goshuyuamide I (**25**), 1-methyl-2-[(*Z*)-5-undecenyl]-4(1*H*)-quinolone (**65**), 1-methyl-2-[(4*Z*,7*Z*)-4,7-tridecadienyl]-4(1*H*)-quinolone (**83**), evocarpine (**73**), and 1-methyl-2-[(6*Z*,9*Z*)-6,9-pentadecadienyl]-4(1*H*)-quinolone (**96**)) [245], and another five quinoline alkaloid (1-methyl-2-undecyl-4(1*H*)-quinolone (**62**),1-methyl-2-[(6*Z*,9*Z*,12*E*)-pentadecatriene]-4(1*H*)-quinolone (**99**), 1-methyl-2-[(*Z*)-7-tridecenyl]-4(1*H*)-quinolone (**80**), dihydroevocarpine (**72**), and 1-methyl-2-tetradecy-4(1*H*)-quinolone (**89**)) [60], are speculated as possible hepatotoxic components based on spectrum-toxicity relationship and UPLC-Q-TOF-MS, whether these components were toxic as well still requires further exploring and researching. Therefore, attention should be given to monitoring bile acid metabolism in the clinical use of Euodiae Fructus.

It was worth noting that P450-mediated dehydrogenation reactions of evodiamine and rutaecarpine might cause toxicities through the generation of highly electrophilic intermediate and lead to drug-drug interactions mainly via the inactivation of CYP3A4 [246], Zhu et al. demonstrated that the induction of cytochrome P450 enzyme genes, hepatic transporters and phase-2 enzyme genes are involved in the interaction between rutaecarpine and drugs [247]. In addition, evodiamine could inhibit CYP1A2, CYP2C9 and CYP2D6 in rats, which might affect the disposition of drugs that rely on these pathways [248]. Therefore, it is necessary to pay attention to CYP3A4-, CYP1A2-, CYP2C9- and CYP2D6-mediated herb–drug interactions between Euodiae Fructus and western drugs to avoid undertreatment.

Additionally, dehydroevodiamine inhibited hERG channels with IC_50_ values of 253.2 ± 26.3 nM on human embryonic kidney cells, prolonged the action potential duration (APD) in human induced pluripotent stem cell-derived cardiomyocytes (hiPSC-CMs) in a concentration-dependent manner from 0.01 to 1 μM and induced early afterdepolarizations (EADs) at 3 μM. Dehydroevodiamine (0.5 mg/kg) induced TdP arrhythmias in 2 out of 8 animals, and STV increased accordingly [249] in rabbits. In another study, evodiamine inhibited rat cardiomyocytes viability with IC_50_ value of 28.44 µg/mL at 24 h, increased LDH release and MDA levels, and reduced superoxide dismutase (SOD) activity on primary cultured neonatal rat cardiomyocytes. In zebrafish model, evodiamine also has a 10% lethal concentration of 354 ng/mL and induce cardiac malfunction, as evidenced by changes in heart rate and circulation, and pericardial malformations. These results indicated that evodiamine could cause cardiovascular side effects involving oxidative stress [250].

Since Euodiae Fructus contains potentially toxic compounds, reliable analytical methods are needed to control the quality of product development to ensure that the potential toxic components of Euodiae Fructus-related products are kept below allowable levels, and more attention should be given to herb–drug interactions and monitoring bile acid metabolism in the clinical use of Euodiae Fructus.

## Quality control

As we all know, the intrinsic quality of TCM might vary greatly due to different geographic conditions and harvest periods [251]. Therefore, an efficient, rapid, sensitive and reproducible detection method was important to ensure the quality of each batch of medicinal materials [252]. According to the 2020 Edition of Chinese Pharmacopoeia, the concentration of evodiamine and rutaecarpine should exceed 0.15%, and the concentration of limonin should exceed 0.20% as determined by HPLC with the mobile phase making up of 0.02% phosphoric acid water and acetonitrile-tetrahydrofuran (25:15) at a ratio of 35: 65, and the detection wavelength should be at 225 nm. However, due to the pharmacological activity and toxicity of multiple ingredients mentioned above, the content of single or small amount of labeled compounds cannot accurately reflect the quality of TCM [253]. With the advancement of analytical tools, it is necessary to adopt more advanced detection methods to qualitatively and quantitatively analyze as many biologically active ingredients as possible. A total of 13 compounds: Wuchuyuamide-I, quercetin, limonin, evodiamine, rutaecarpine [254], dehydroevodiamine, evodine [26], evodiamide, 14-formyldihydrorutaecarpine [25], 1-methyl-2-undecyl-4(1H)quinolone, evocarpine, 1-methy-2-[(6*Z*,9*Z*)]-6,9pentadecadienyl-4-(1H)-quinolone, and dihydroevocarpine [255], were selected to ensure the quality of Euodiae Fructus by HPLC–DAD, HPLC–DAD-MS/MS, HPLC/UV/APCI-MS/MS, and CEC-MS, and the additional details are listed in Table 6. To evaluate the quality, the newly established fingerprint analysis was conducted on this kind of plants. The fingerprint analysis of Euodiae Fructus was carried out and the results suggest that the chemical components would vary greatly in different locations and vary a little in different years in the same site [64, 256]. In recent years, one study compared the differences of essential oils from three species of Euodiae Fructus cultured in China. The results showed that the differences in chemical composition and oil production within species are greater than the differences between species [91].

**Table 6:** Quantitative analysis for the quality control of Euodiae Fructus

Analytes	Method	Results	References
Wuchuyuamide-I, Quercetin, Limonin, Evodiamine, Rutaecarpine	HPLC–DAD	0.0059–0.0563%, 0.0045–0.2144%, 0.1186–2.3036%, 0.0053–0.7957% and 0.0222–0.6236%, respectively (contents)	[254]
Dehydroevodiamine, Evodine, Evodiamine and Rutaecarpine	HPLC–DAD-MS/MS	0.10–0.51%, 0.49–3.12%, 0.07–1.56%, and 0.10–0.69%, respectively (contents)	[26]
Rutaecarpine, Evodiamine, Evodiamide, 14-Formyldihydrorutaecarpine, Dehydroevodiamine	HPLC/UV/APCI-MS/MS	0.061–0.550 mg/g, 0.039–1.623 mg/g, 0.0037–0.455 mg/g, 0.024–0.065 mg/g, 0.122–0.863 mg/g and 0.0069–0.741 mg/g, respectively(contents)	[25]
Limonin, Evodiamine, Rutaecarpine, 1-Methyl-2-undecyl-4(1*H*) quinolone, Evocarpine, 1-Methy-2-[(6*Z*,9*Z*)]-6,9-pentadecadienyl-4-(1*H*)-quinolone and Dihydroevocarpine	HPLC–DAD	1.129–13.478%, 0.078–2.070%, 0.157–1.127%, 0.055–0.591%, 0.100–1.881%, 0.153–1.273%, and 0.083–0.592%, respectively (contents)	[255]
Limonin, Evodiamine, Rutaecarpine	CEC-MS	0.24–0.31 μg/g, 0.15–1.2 μg/g and 0.16–0.6 μg/g, respectively (contents)	[257]
Fingerprint	HPLC–ESI-MS^n^	A total of 25 common peaks were found in the HPLC fingerprints of Evodiae Fructus	[64]
Fingerprint	HPLC	A total of 20 major common peaks were found in the HPLC fingerprints of Evodiae Fructus	[256]
Essential Oils	GC/MS	A total of 79 compounds were identified from the nearly mature fruits of Evodiae Fructus, accounting for 75.86–99.11% of the total oils	[91]

## Conclusions

This review has summarized the multifaceted uses and recent findings regarding studies of the phytochemistry, traditional use, bioactive constituents, pharmacology, toxicity, and quality control of different extracts and compounds of Euodiae Fructus and provides a practical base for further scientific research and favorable clinical application on this plant. Extensive researches have been conducted on the phytochemistry of the Euodiae Fructus and approximately 240 compounds have been isolated and identified from this plant, including alkaloids, terpenoids, steroids, phenols, volatile oil and other compounds. As the literature has demonstrated, alkaloids and terpenoids are the main components of Euodiae Fructus, and alkaloids are mostly responsible for its pharmacological activities. Additionally, recent reports have primarily focused on evaluating anticancer, antibacterial, anti-inflammatory, insecticidal, anti-cardiovascular disease, neuroprotective, anti-obesity and anti-diabetic activities of the herbal medicines derived from this plant. In particular, the indole alkaloids (e.g., evodiamine, rutaecarpine and dehydroevodiamine) and limonin have been confirmed to has low toxicity and high medicinal value through various pharmacological activities in vivo and in vitro investigations.

Euodiae Fructus exhibits a diverse set of pharmacological properties and its chemistry is complex. For these reasons, it is of great importance to systematically and critically evaluate the future direction and application of this field. Although many efforts have been made to study these plants, there are also a number of points and aspects that need to be improved and researched further: (1) According to TCM, Euodiae Fructus is traditionally considered to have mild toxicity, and a few support studies have been linked to its toxicity, including the potential hepatotoxicity, CYP inhibition, and cardiotoxicity of this plant. Thus, it is necessary to investigate the potential toxic effects induced by Euodiae Fructus and clarify the toxic components, target-organs and mechanisms, so as to lay a foundation for future research. (2) Several traditional uses of these plants have been validated in recent pharmacological studies, but some of these were only tested in vitro. Therefore, the effectiveness of these compounds in vivo and comprehensive placebo-controlled and double-blind clinical trials need to be further studied, and more detailed pharmacology and mechanism of action may help to better understand TCM theory. (3) Alkaloids are traditionally considered as the major bioactive compounds in Euodiae Fructus. However, their mechanisms of action remain unclear, and further studies are required to understand the structure–activity relationships of these constituents and bioactivities. For isolated alkaloids, too many researches are focused on evodiamine and rutaecarpine, and there are other active ingredients like dehydroevodiamine, evocarpine and dihydroevocarpine, etc. that have been lacked of research or ignored. Further investigation should be encouraged to study these components or their analogues. (4) Numerous studies have demonstrated evodiamine process extensive activities, however, due to its poor water solubility and low oral bioavailability, thereby limiting its anticancer efficacy clinically. Future studies should aim to overcome these problems in the clinical application of TCM. (5) In view of the toxicity of some compounds, reliable analytical methods are required for proper quality control of drug development to ensure that potential toxic components remain below the tolerance level of Euodiae Fructus.

## Data Availability

The datasets generated during and/or analysed during the current study are available from the corresponding author on reasonable request.
